# Metabolomics Characterization of Scleractinia Corals with Different Life-History Strategies: A Case Study about *Pocillopora meandrina* and *Seriatopora hystrix* in the South China Sea

**DOI:** 10.3390/metabo12111079

**Published:** 2022-11-08

**Authors:** Jiying Pei, Shiguo Chen, Kefu Yu, Junjie Hu, Yitong Wang, Jingjing Zhang, Zhenjun Qin, Ruijie Zhang, Ting-Hao Kuo, Hsin-Hsiang Chung, Cheng-Chih Hsu

**Affiliations:** 1 Coral Reef Research Center of China, Guangxi Laboratory on the Study of Coral Reefs in the South China Sea, School of Marine Sciences, Guangxi University, Nanning 530000, China; 2 Southern Marine Science and Engineering Guangdong Laboratory, Zhuhai 519080, China; 3 Department of Chemistry, National Taiwan University, Taipei 10617, Taiwan

**Keywords:** life-history strategy, metabolomics, mass spectrometry, *Pocillopora meandrina*, *Seriatopora hystrix*

## Abstract

Life-history strategies play a critical role in susceptibility to environmental stresses for Scleractinia coral. Metabolomics, which is capable of determining the metabolic responses of biological systems to genetic and environmental changes, is competent for the characterization of species’ biological traits. In this study, two coral species (*Pocillopora meandrina* and *Seriatopora hystrix* in the South China Sea) with different life-history strategies (“competitive” and “weedy”) were targeted, and untargeted mass spectrometry metabolomics combined with molecular networking was applied to characterize their differential metabolic pathways. The results show that lyso-platelet activating factors (lyso-PAFs), diacylglyceryl carboxyhydroxymethylcholine (DGCC), aromatic amino acids, and sulfhydryl compounds were more enriched in *P. meandrina*, whereas new phospholipids, dehydrated phosphoglycerol dihydroceramide (de-PG DHC), monoacylglycerol (MAG), fatty acids (FA) (C < 18), short peptides, and guanidine compounds were more enriched in *S. hystrix*. The metabolic pathways involved immune response, energy metabolism, cellular membrane structure regulation, oxidative stress system, secondary metabolite synthesis, etc. While the immune system (lysoPAF) and secondary metabolite synthesis (aromatic amino acids and sulfhydryl compounds) facilitates fast growth and resistance to environmental stressors of *P. meandrina*, the cell membrane structure (structural lipids), energy storage (storage lipids), oxidative stress system (short peptides), and secondary metabolite synthesis (guanidine compounds) are beneficial to the survival of *S. hystrix* in harsh conditions. This study contributes to the understanding of the potential molecular traits underlying life-history strategies of different coral species.

## 1. Introduction

The coral reef ecosystems possess the highest biodiversity and productivity. They provide critical marine resources (including fishery, coastal protection, tourism, and biodiversity) for the economic and social development of humankind. The coral community is composed of various species with different life-history strategies, and the life-history composition of coral taxa affects the capacity of the coral community to cope with climate change and human pressure [1]. Emily S. Darling et al. divided coral’s life-history strategies into four categories: competitive, weedy, stress-tolerant and generalist taxa based on their biological traits (colony morphology, growth, reproductive mode, and et al.) [2]. A competitive life-history strategy is typically efficient at using resources and can dominate communities in stable or ideal environments, such as shallow and high light environments. The species with the competitive life-history strategy usually have A branching growth form, fast growth rate, and broadcast spawning reproductive mode. Comparatively, the species with weedy life-history strategies generally have smaller colony sizes and a brooding reproductive mode. They can persist in unfavorable and variable environments, and opportunistically colonize a variety of disturbed environments, such as heavily fished reefs or shallow back reef lagoons. Though several previous investigations have proven that chemicals have indeed participated in the regulation of coral’s life-history [3,4], the present investigation of coral’s life-history strategy is more focused on the influencing processes of ecological [5] and environmental [6,7,8,9] conditions, and the prediction of life-history strategy-specific population dynamics [1]. However, the potential molecular traits underlying life-history strategies were seldomly explored.

The coral reefs in the South China Sea (SCS) are located at the northern edge of the “coral triangle” in Southeast Asia. It covers an area of approximately 38,462 km^2^ [10], and accounts for 5% of the world’s coral reefs. The potential economic value created by coral reefs in the SCS is evaluated to be RMB 156.5 × 10^8^ Yuan/year [11]. In the context of global climate change and intensified human activities, the live coral cover (LCC) in the SCS has decreased from 90% at the beginning of the 1970s to 16.3% in 2015 [12], accompanying an estimated economic loss of RMB 258.8 × 10^8^ Yuan in 20 years since 2014 [11]. The transformed ecological status of coral reef communities in the SCS violates the United Nations Sustainable Development Goals. The decrease of LCC may be affected by the composition of the life-history strategies of the coral reef community. For example, *Pocillopora meandrina,* with the life-history strategy of “competitive”, is the dominant species in multiple island-reefs in the SCS [13,14]. It flourishes in an ideal and productive environment, such as the outer reef slope. However, this coral species is sensitive to environmental change. Climate change and *anthropogenic* activities have resulted in the decrease of the LCC of *P. meandrina* worldwide [15]. By comparison, *Seriatopora hystrix,* with the life-history strategy of “weedy”, was nondominant species in all the islands in the SCS. Though *S. hystrix* was seldomly found on outer reef slopes, it can been found in lagoons which are generally unfavorable for coral growth in high temperatures. The previous investigation also indicated that “locally extinct” *S. hystrix* was found in a mesophotic coral ecosystem on Okinawa Island [16]. In terms of phylogenetic relationships, both *P. meandrina* and *S. hystrix* belong to the Pocilloporidae family. While these two species share some similarities in biological traits, such as branching morphology, there exist some discrepancies. While the reproductive pattern of *P. meandrina* is broadcast spawning, that of *S. hystrix* is brooding [17]. The growth rate of *P. meandrina* is faster than that of *S. hystrix* [18].

Life-history strategies are determined by genetic and environmental factors. Metabolomics can be regarded as the ultimate response of biological systems to genetic, transcriptomic, or environmental changes. Thus, it provides a molecular biological method for the characterization of an organism’s life-history strategy. Metabolomics has been intensively used to investigate Scleractinia coral’s response and adaptation mechanism to environmental stresses. For example, Roach et al. analyzed the metabolome difference of historically bleached and unbleached Montipora capitate (in pairs of adjacent corals, where one colony in the pair historically bleached and recovered while the other did not bleach). They found that the content and degree of unsaturation of diacylglyceryl carboxyhydroxymethylcholine (DGCC) were closely related to the historical bleaching phenotypes [19]. Amanda Williams et al. identified several dipeptides as early warning biomarkers of the bleaching of *Montipora capitate* and *Pocillopora acuta* by untargeted metabolomics [20].

Mass spectrometry is one of the most commonly used methods for metabolomics analysis. It possesses the advantages of high sensitivity, label-free features, simultaneous determination of multiple metabolites, and the availability of compound identification. However, compound identification is compromised by the limited mass spectral libraries containing only a fraction of tandem mass spectra (MS/MS spectra) of endogenous metabolites. Molecular network technologies, such as global natural product social molecular networking (GNPS) and metabolite identification and dysregulated network analysis (MetDNA) [21], are particularly efficient tools for processing and annotating MS/MS spectra through spectral similarity algorithms, which significantly saves data interpretation time and accelerates the identification of unknown compounds [22,23,24]. Thereinto, GNPS has been extensively applied in the characterization and discovery of unknown metabolites [25], and the complementary tool of feature-based molecular networking (FBMN) further enhances the quantitative analyses and isomer distinction in GNPS [24]. Here, we combined untargeted metabolomics and FBMN to explore the underlying molecular traits that contribute to the different life-history strategies of *P. meandrina* and *S. hystrix* in the SCS, and to analyze the potential biological functions of the differential metabolites.

## 2. Materials and Methods

### 2.1. Study Site and Sample Collection

*P. meandrina* and *S. hystrix* were sampled from the outer reef slope and lagoon of Panshi atoll (N16°02′~N16°04′, E111°76′~E111°82′), respectively, located in the Xisha island of SCS, on 15 March 2019. The Sea Surface Temperature (SST) fluctuation was higher in the lagoon than in the outer reef slope. The monthly SSTs of the outer reef slope and lagoon in 2019 were recorded to be 28.2 ± 0.5 °C and 30.2 ± 0.3 °C respectively. The salinity, nutrient availability, and turbidity were all higher in the lagoon. In contrast, the transparency of the lagoon was noticeably lower than that of the outer reef slope. The detailed water quality parameters are referred to in the literature [26]. In this study, eight *P. meandrina* fragments were collected in the outer reef slope of Panshi atoll and four *S. hystrix* fragments were collected in the lagoon. After collection, the coral fragments were transferred to −20 °C refrigeration by a bucket containing seawater for subsequent analysis.

### 2.2. Metabolites Extraction from Coral Fragments

Coral tissue was washed off from the coral skeleton by a water toothpick with chilled ultrapure milli-Q water. After lyophilization, 10 mg of dried coral tissue powder containing an internal standard (caffeine-D9, 3 μg) was extracted by 0.5 mL of ice-cold methanol/water (*v*/*v*, 7:3). The extract was centrifuged at 10,000× *g* for 5 min at 4 °C. The supernatant was withdrawn and stored temporarily on dry ice. The remaining coral tissue was repeatedly extracted with the method above twice and the three extracts were combined. After filtration with a 0.22 μm nylon syringe filter, the extracts were stored at −80 °C until liquid chromatography–tandem mass spectrometry (LC–MS/MS) analysis. The experimental procedures are referred to in Figure S1.

### 2.3. Mass Spectrometry Data Collection and Pre-Processing

The extracts were analyzed on a Thermo^TM^ Q-Exactive^TM^ mass spectrometer coupled to a Dionex UltiMate 3000 UHPLC system. The mobile phase was composed of water with 0.1% formic acid and methanol with 0.1% formic acid. The chromatographic separation was performed at 30 °C using an ACQUITY CSH C_18_ column (2.1 × 100 mm; 1.7 μm; Waters, MA, USA) and a flow rate of 200 μL/min. The percentage of mobile phase B was set as follows: 0–3 min, 5%; 3–20 min, 5–95%; 20–25 min, 95%; 25–26 min, 95–5%; 26–30 min, 5%. The injection volume of each sample was 2 µL. Data were collected in positive electrospray ionization mode with the data-dependent acquisition (DDA). A DDA method was performed by an alternating collection of full mass spectra from *m/z* 100 to 1000 and MS/MS spectra of the top-10 most intense compounds.

The chromatographic features were extracted from the raw mass spectrometry data using open-source MZmine software (T. Pluskal, S. Castillo, A. Villar-Briones, M. Orešič, 2010; version 2.51 http://mzmine.github.io/( accessed on 1 August 2021)). The feature extraction procedure included 11 steps: MS and MS/MS mass detection, chromatogram builder, chromatogram deconvolution, isotopic peak grouper, join aligner, feature list rows filter, peak finder, duplicate peak filter, adduct search, and fragment search. The detailed parameters for each step are provided in Table S1. Finally, the signal intensities of all metabolites were normalized by the signal intensity of the internal standard, caffeine-D9.

### 2.4. Statistical Analysis

The exported chromatographic feature table was introduced to SIMCA-P (v14.1, Umetrics, Umea, Sweden) for principal component analysis (PCA) and orthogonal partial least square discriminant analysis (OPLS-DA). All data were unit variance- and Pareto-scaled before PCA and OPLS-DA analysis, respectively. Variable Importance in the Projection (VIP) > 1.0 is supposed to contribute significantly to the separation of two groups. The Shapiro–Wilk test was used to test the normality of a set of data before statistical significance analysis. An independent sample *t*-test was applied when the data were normally distributed; otherwise, a non-parametric test was used. The *p*-value lower than 0.05 was regarded as significant, whereas *p* < 0.01 was considered highly significant.

### 2.5. Molecular Network Analysis and Metabolic Pathway Analysis

A mgf-formatted file and a feature quantification table exported from MZmine software were uploaded to the GNPS website for running FBMN workflow. FBMN was performed with parent and fragment mass ion tolerances of 0.02 Da, a cosine score of 0.7, minimum matched peaks of 2, library search minimum matched peaks of 2, a library search score threshold of 0.7, and a minimum peak intensity of 20,000. The feature-based molecular networking job is available at: https://gnps.ucsd.edu/ProteoSAFe/status.jsp?task=ae3692556f2649e1904cd23f0b04928c (accessed on 5 August 2021). The FBMN output files were visualized using Cytoscape software version 3.8.0 (https://cytoscape.org/index.html (accessed on 5 August 2021)).

The metabolic pathway analysis was performed by Metaboanalyst version 5.0 (https://www.metaboanalyst.ca/(accessed, accessed on 5 August 2021)) [27] and the KEGG metabolic pathway database (https://www.genome.jp/kegg/pathway.html (accessed on 5 August 2021)).

### 2.6. Data Quality Control

Quality control (QC) was conducted throughout the study. QC samples were prepared by pooling an equal quantity of each coral tissue dry powder, and extracted by the same method as the *P. meandrina* and *S. hystrix* samples. To monitor the stability and reproducibility of instrument analysis, QC samples were injected five times in the beginning and analyzed for every three samples. A blank solution, which matched the composition of the extraction solvent, was injected before the QC sample to assess the background signal and carryover during analysis. Only metabolites detected in QC samples with a coefficient of variation (CV) < 30% were kept for further analysis. All metabolites were identified with errors within five parts per million (ppm) mass accuracy.

## 3. Results

### 3.1. Chemical Diversity and Multivariate Statistical Analysis of the Metabolites of P. meandrina and S. hystrix

**Figure 1:**
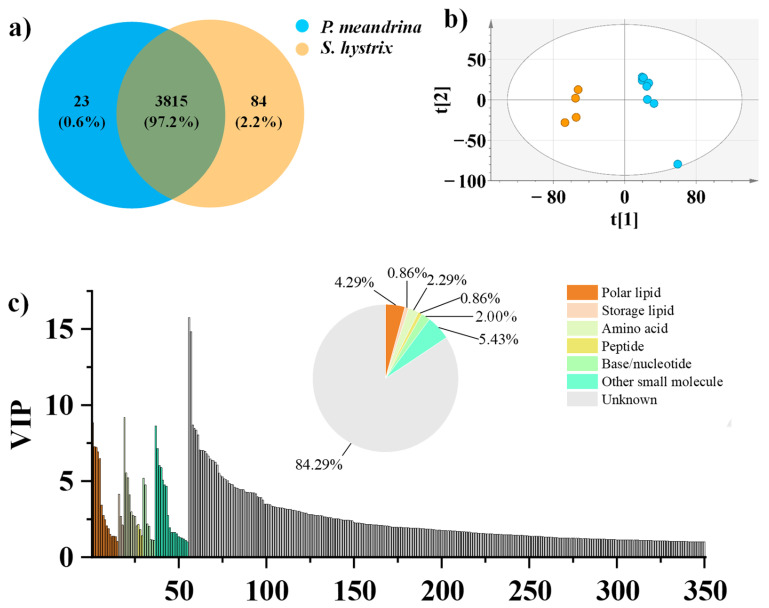
(**a**) Venn diagram of the metabolites of *P. meandrina* and *S. hystrix*. (**b**) PCA plot for differentiating *P. meandrina* and *S. hystrix*. (**c**) Categorization of the metabolites (VIP > 1, *p* < 0.05) making contributions to the differentiation of *P. meandrina* and *S. hystrix*. The color legend is for both the pie chart and bar plot.

To comprehensively profile the metabolome of *P. meandrina* and *S. hystrix,* the tissues of eight *P. meandrina* and four *S. hystrix* were parallelly extracted and analyzed by LC–high resolution mass spectrometry. The feature peaks of LC were extracted by an open source software, Mzmine. Three thousand eight hundred and fifteen metabolite feature peaks were extracted from the metabolomics dataset of *P. meandrina* and *S. hystrix*. It is noteworthy that the selected extraction protocol and mass spectrometry detection method confined the molecular coverage to polar and semi-polar compounds, because the extraction solvent of methanol/water (*v*/*v*, 7:3) used in this study is inefficient for extracting non-polar and weak polar compounds, such as terpene. To maximize the ionization efficiency of extracted metabolites, an ESI source was used in mass spectrometry. To more comprehensively understand the metabolite information, the coral tissue should be multiply extracted by solvents with gradient polarities, and additional mass spectrometry instruments, including GC–MS and LC-APCI–MS, should be employed in future investigation. The signal intensities of 2044 out of the 3815 metabolites (accounting for 53.6%) were higher in *P. meandrina*. Correspondingly, the signal intensities of 1771 metabolites, accounting for 46.4% of the total metabolites, were higher in *S. hystrix*. Especially, 23 metabolites were unique to *P. meandrina* and 84 metabolites were unique to *S. hystrix* (Figure 1a). An index based on the formula of Shannon Index (H = −ΣPilog_2_Pi, where Pi is the ratio of the signal intensity of one metabolite to the sum of the signal intensities of all metabolites in one sample) was calculated to compare the chemical diversity of the two corals; it showed that the chemical diversity of *P. meandrina* was higher than *S. hystrix*, but there was no significant difference (Figure S2).

Multivariate statistical analyses, including unsupervised PCA and supervised OPLS-DA, were performed to better visualize the subtle differences in the metabolome between *P. meandrina* and *S. hystrix*. The PCA result showed that all *P. meandrina* and *S. hystrix* samples were within the 95% Hotelling’s T-squared ellipse and significantly separated into clusters (Figure 1b). The first principal component (PC1) and second principal component (PC2) explained 30.5% and 15.4% of the total variance of all samples respectively. The OPLS-DA result also showed complete segregation between the metabolomic profiles of *P. meandrina* and *S. hystrix* (Figure S3a). To assess the OPLS-DA model’s predictive accuracy, seven-fold internal cross-validation was conducted. The parameters of the model’s predictive accuracy were R^2^X_cum_ = 0.538, R^2^Y_cum_ = 1, and Q^2^Y_cum_ = 0.973. Two hundred times permutation test shows that the OPLS-DA model predicts well without overfitting (Figure S3b). The important projection value (VIP) reflects the importance of variables to the OPLS-DA model. The metabolites responsible for the separation between *P. meandrina* and *S. hystrix* were screened according to the criteria of VIP > 1 and *p* < 0.05 (p, the corrected p values from Student’s *t*-test). To summarize, 350 metabolites were screened out as the differential metabolites between *P. meandrina* and *S. hystrix* (Figure 1c). Among them, 160 metabolites were more abundant in *P. meandrina*, and 190 metabolites were more abundant in *S. hystrix*.

### 3.2. Identification of the Differential Metabolites between P. meandrina and S. hystrix

To annotate the chemical identification of metabolites, the metabolomics dataset of *P. meandrina* and *S. hystrix* was processed through the FBMN online infrastructure. FBMN aims to annotate unknown metabolites through annotated metabolites within the same sub-network based on spectral similarity. Additional algorithms, such as Network Annotation Propagation (NAP) [28] and MetDNA [21], constructed the molecular network by incorporating molecular similarity from in silico-predicted MS/MS spectra and metabolic reaction information, respectively. These algorithms do not necessitate the annotation of unknown metabolites from the standard spectral library. However, they may sacrifice certain parameters in terms of annotation accuracy, annotated metabolite numbers or unavailability for natural product discovery. In our study, the resulting molecular networking from FBMN encompassed hundreds of molecular clusters. Two hundred and thirteen metabolites were annotated by searching against the GNPS database (level two or three, according to previously published standards) [29]. To speculate on the identities of the unknown differential metabolites, the MS/MS spectra in the molecular networking were sequentially thoroughly inspected. In a molecular network, each node represents a consensus MS/MS spectrum of a metabolite. When the similarity score between two nodes is greater than the threshold cosine value, the two nodes will be connected to form a cluster. The number of identified metabolites was extended to 286 by the further inspection through manual analysis. Totally, we successfully identified 55 differential metabolites including the categories of fatty acids (FA) and lipids, small peptides, amino acids, nucleotides, and other small molecules (Figure S4). Their locations in the molecular network, mass spectrometric characteristics, and abundance comparison in *P. meandrina* and *S. hystrix* are detailed in the following.

#### 3.2.1. Lipid and FA

**Figure 2:**
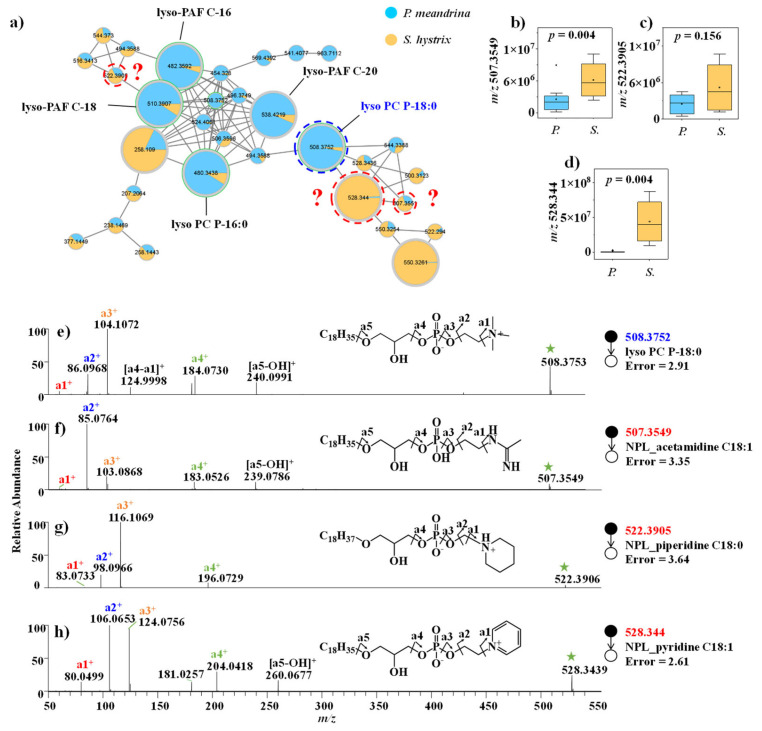
(**a**) Molecular networking of phospholipid family. (**b**–**d**) Comparison of the signal intensities of the phospholipid family between *P. meandrina* and *S. hystrix* and (**e**–**h**) their corresponding mass spectra. (The green asterisk means the parent ion of the MS/MS spectrum. It is also applicable to other figures. The red query means unannotated compound in the GNPs library.)

Multiple phosphatidylcholines (PC) with VIP > 1, such as lyso-platelet-activating factor (lyso-PAF) C-16, lyso-PAF C-18, lyso-PAF C-20, lyso PC P-16:0 and lyso PC P-18:0, were annotated through the spectral library searching from GNPS database. Their signal intensities were higher in *P. meandrina* (Table S2). Notably, their adjacent nodes with *m/z* 507.3549, 522.3905, and 528.3439 in the same molecular family were detected with higher signal intensities in *S. hystrix* (Figure 2a–d, Table S3). To speculate on the identities of these unknown structural analogues as phosphatidylcholine, their MS/MS spectra were carefully inspected. The fragment ions of *m/z* 86.0968, 104.1072, 124.9998, 184.0730, and 240.0991 from lyso PC P-18:0 (*m/z* 508.3752) indicated a glycerophosphoryl choline group (Figure 2e). By comparison, the characteristic fragment ions of the nodes with *m/z* 528.3439, 507.3549, and 522.3905 shared a similar dissociation pattern as lyso PC P-18:0 (Figure 2f–h). For example, the characteristic fragment ions of the node with *m/z* 507.3549 were *m/z* 85.0764, 103.0868, 183.0526, and 239.0786 (Figure 2f), with a mass shift of 1.0204 Daltons from the fragment ions (*m/z* 86.0968, 104.1072, 184.0730, and 240.0991) of lyso PC P-18:0 (Figure 2e). Based on the elemental compositions predicted by high-resolution *m/z* values and the fragment pattern, this unknown metabolite was identified as acetamidine head group-substituted phosphatidylcholine. Similarly, the mass shift of 11.9997 Daltons of the fragment ions derived from the parent ions *m/z* 508.3752 (lyso PC P-18:0) and *m/z* 522.3905 (unknown) indicated that the head group in the unknown metabolites was piperidine, compared to a quaternary ammonium group in lyso PC P-18:0 (Figure 2g). Since the mass difference between the parent ions was 14.0153 Daltons, the fatty acid carbon chain in the unknown phospholipid was inferred to be C_18_H_37_. The mass shift of 19.9684 Daltons of the fragment ions derived from parent ions *m/z* 508.3752 (lyso PC P-18:0) and *m/z* 528.344 (unknown) indicated that the head group in the unknown metabolites was pyridine (Figure 2h). Nonetheless, we failed to dereplicate these phosphatidylcholines by searching against most online databases, including GNPS, MassBank, METLIN, ChemSpider, and Lipid-Maps, and via a literature search (Figure S5), which means that these novel metabolites were reported for the first time.

**Figure 3:**
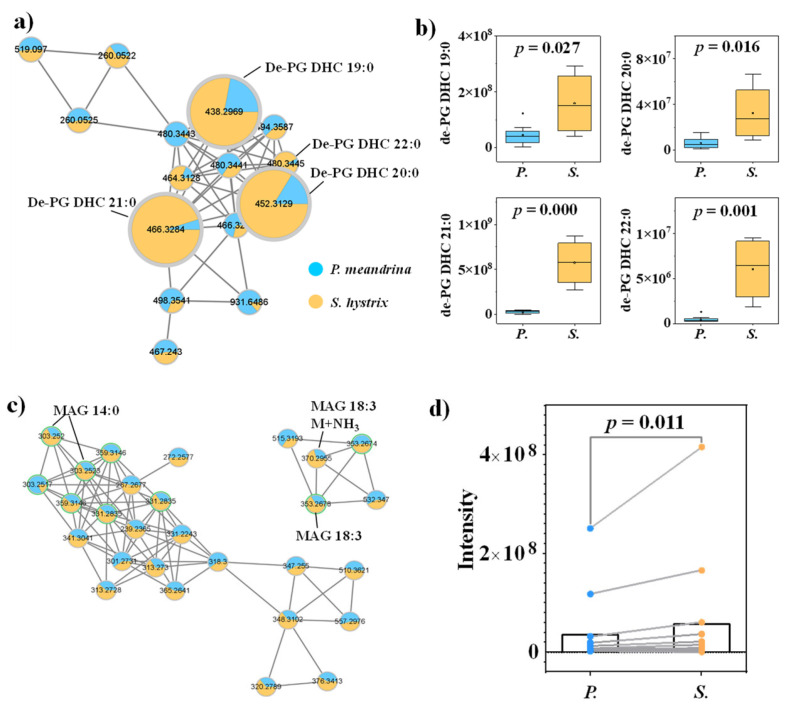
(**a**) Molecular networking of de-PG DHC family and (**b**) the corresponding box diagrams of the signal intensities between *P. meandrina* and *S. hystrix*. (**c**) Molecular networking of MAG family and (**d**) matched samples t-test between the signal intensities of *P. meandrina* and *S. hystrix*. (The green asterisk means the parent ion of the MS/MS spectrum.)

Other lipids and FAs, including dehydrated phosphoglycerol-dihydroceramide (de-PG DHC), lyso-DGCC and monoacylglycerol (MAG), were also annotated from the molecular networking. On average, the signal intensities of de-PG DHC and MAG were higher in *S. hystrix* (Figure 3, Figures S6 and S7), and the signal intensities of DGCC were higher in *P. meandrina* (Figure S8). Though the relatively short-chain FAs (C < 18) were more enriched in *S. hystrix*, the relatively long-chain FAs (C ≥ 18) were more enriched in *P. meandrina* (Figure S9). Furthermore, we calculated the degree of unsaturation of all the identified structural lipids (de-PG DHC, lyso-DGCC, PC, phosphatidylethnolamine and new lipids). The unsaturation index (UI) was calculated using the following formula:

$$\mathrm{UIx}=\left[\sum y\left({\%\mathrm{lipid}}_{y}\times {\mathrm{total}\;\mathrm{number}\;\mathrm{of}\;\mathrm{double}\;\mathrm{bonds}\;\mathrm{lipid}}_{y}\right)\right]/100$$

where *y* is every molecular lipid species belonging to the lipid class *x*. The different compositions of structural lipids between the taxa resulted in a lower UI in *S. hystrix* as compared to *P. meandrina* (0.007 vs. 0.014).

#### 3.2.2. Small Peptide

**Figure 4:**
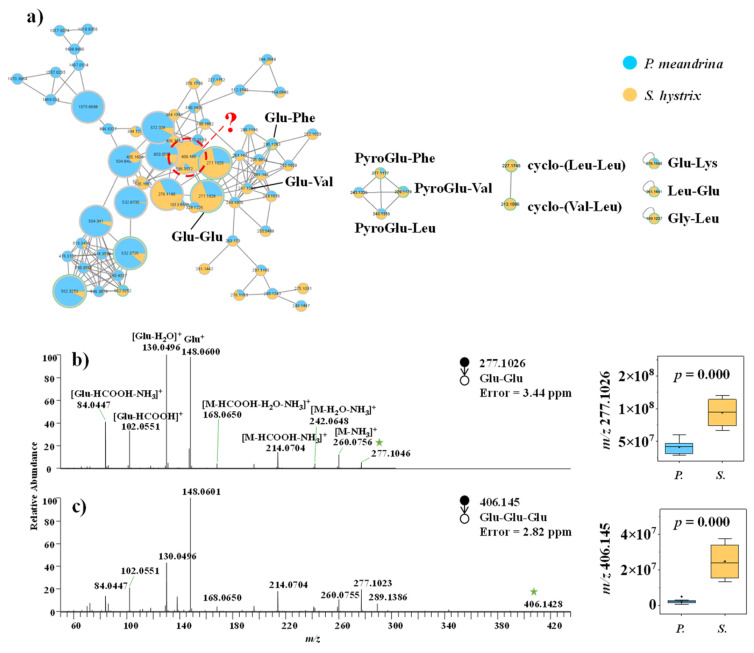
(**a**) Molecular networking of small peptide family. (**b**,**c**) Mass spectra of small peptides and the corresponding box diagrams of the signal intensities between *P. meandrina* and *S. hystrix*. (The green asterisk means the parent ion of the MS/MS spectrum. It is also applicable to other figures. The red query means unannotated compound in the GNPs library.)

Dereplication through comparing the MS/MS spectra against spectral libraries at GNPS gave the annotation of multiple dipeptides (Figure 4a), including Glu–Glu (Figure 4b), Glu–Lys, Leu–Glu, Gly–Leu, cyclo-(Val–Leu), cyclo-(Leu–Leu), PyroGlu–Val, PyroGlu–Phe, and Glu–Phe (Figure S10). Based on the MS/MS spectral similarity and representative MS/MS fragments indicative of the molecular weights of amino acid residues, Glu–Glu–Glu (Figure 4c), Glu–Val, and PyroGlu–Leu (Figure S10) were putatively annotated from the molecular networking. Most of the identified short peptides were more accumulated in *S. hystrix* (Table S4).

#### 3.2.3. Other Small Molecules

Besides lipids, FAs, and small peptides, other small molecules, including amino acids, base/nucleotide, carnitine, and guanidyl compounds, were also annotated from the GNPS libraries. Among these small molecular metabolites, *P. meandrina* accumulated higher levels of tyrosine, phenylalanine, tryptophan, deoxyadenosine monophosphate (DAMP), thymidine monophosphate, 5’-methylthio-adenosine, succinyl–adenosine, propionyl–carnitine, alanopine, 1-carboxyethyl–isoleucine, S-(5’-Adenosyl)-L-homocysteine, lysine, methionine, and 4-hydroxy-D-proline, whereas *S. hystrix* accumulated higher levels of adenine, thymine, (4-carboxy-2-hydroxybutyl)-trimethylazanium, isovaleryl–carnitine, arginine, 4-guanidinobutyric acid, indole-3-carboxaldehyde, 3-indolepropionic acid, saccharopine, creatine, and maltose (Table S5, Figure S11). It is noteworthy that tyrosine, phenylalanine, and tryptophan all belong to the aromatic amino acid family, and they were consistently more abundant in *P. meandrina*.

**Figure 5:**
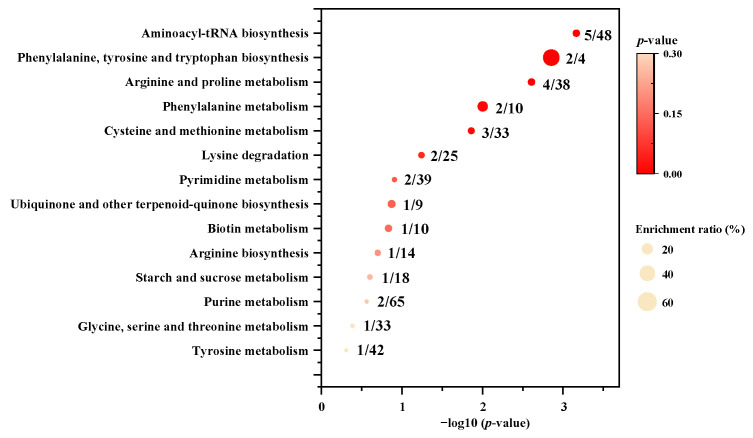
Metabolic pathway enrichment analysis between *P. meandrina* and *S. hystrix*. The numbers before and after the oblique line represent observed differential metabolites and total metabolites in the metabolic pathway, respectively.

The KEGG pathway enrichment analysis of the 25 differentially small molecular metabolites was performed by MetaboAnalyst 5.0 to identify the differential metabolic pathways between *P. meandrina* and *S. hystrix*. The results showed that fourteen differentially enriched metabolic pathways were involved (Figure 5), among which five had *p*-values less than 0.05. These significant differential metabolic pathways are aminoacyl–tRNA biosynthesis, phenylalanine, tyrosine and tryptophan biosynthesis, arginine and proline metabolism, phenylalanine metabolism, and cysteine and methionine metabolism. Therein, aminoacyl–tRNA biosynthesis involved five differential amino acids (phenylalanine, arginine, methionine, lysine, and tyrosine). Phenylalanine, tyrosine and tryptophan biosynthesis, and phenylalanine metabolism involved two differential aromatic amino acids (phenylalanine, and tyrosine). The arginine and proline metabolism involved three differential guanidyl compounds (arginine, 4-guanidinobutanoic acid, and creatine) as well as 4-hydroxy-D-proline. Cysteine and methionine metabolism involved three differential sulfhydryl metabolites (5’-methylthio-adenosine, methionine, S-(5′-adenosyl)-L-homocysteine). In summary, all of the five metabolic pathways belong to amino acid metabolism.

## 4. Discussion

Metabolites are the end products of cellular regulation processes, and play an important role in energy metabolism, immune regulation and oxidative stress. The abundance of metabolites can indicate the physiological status of organisms. For example, when *Montipora capitata* is preparing to spawn, it metabolizes more montiporic acids [20]. When the growing habitat of *Sarcophyton ehrenbergi* changes from wild to an aquarium, it metabolizes less cembranoids [30]. In our study, the contents of a variety of metabolites, including lipids, FAs, amino acids, nucleotides, and other small molecules, were observed to differ between *P. meandrina* and *S. hystrix*. Their potential biological functions for the formation of the life-history strategies of *P. meandrina* and *S. hystrix* will be discussed in detail subsequently.

### 4.1. Regulation of Immune Response, Cellular Membrane Structure and Energy Metabolism by Lipids and FAs

Structural lipids participate in various physiological processes of organisms, such as immune regulation [31], construction of cell membrane, and signal transduction [32]. Platelet-activating factor (PAF) and lyso-PAF, two central inflammatory modulators identified in Scleractinia coral, belong to the phospholipid of structural lipids, and closely related to cell growth and tissue repair. It was reported that rapid tissue growth and exposure to stressful environments, such as competitive growth habitat or intensive ultraviolet radiation, stimulated coral to produce more PAF [33]. In our investigation, the higher abundance of lyso-PAF in *P. meandrina* may be beneficial to its competition with other species and rapid tissue repair after injuries, which results in the faster growth rate.

The content and degree of unsaturation of structural lipids affect the melting point of cell membranes, which further affects their physiological characteristics, such as fluidity [34]. It is reported that thermo-tolerant Symbiodiniaceae, *Durusdinium trenchii*, contained a significantly lower amount of DGCC than thermo-sensitive *Cladocopium* C3 [35], and the degree of unsaturation of DGCC was significantly lower in historically non-bleached corals than in historically bleached ones [19]. Multiple field investigations show that *S. hystrix* was more opportunistic in accommodating temperature oscillation. For example, in the context of global climate change, the growth rate of *Pocillopora* genus in Lord Howe island decreased by 30% from 1994/1995 to 2010/2011. By comparison, the growth rate of *S. hystrix* was hardly affected [36]. Furthermore, by inspecting the growth habitats of *P. meandrina* and *S. hystrix* in the SCS, *P. meandrina* generally lives in the outer reef slopes with strong water exchange and tiny temperature fluctuations, whereas *S. hystrix* was frequently found in lagoons with weak water exchange and strong temperature fluctuation in a day. In our investigation, the lower content of DGCC and lower UI of structural lipids in *S. hystrix* may serve to acclimate to high temperature environments by adjusting cell membrane structure. Previous investigations showed that *Seriatopora caliendrum* could adapt to the upwelling region with strong temperature oscillations by adjusting the content and saturation of phosphatidylcholines and plasmanylcholines [37]. As for the biological functions of the newly identified phospholipids, they is still unknown. However, as the constituents of phospholipids, they may also participate in the construction of cell membranes.

The function of storage lipids, such as MAG and FA, is to provide energy for organisms. The lipid analysis of *Pocillopora damicornis* larvae with different life histories showed differential responses to environmental change [38]. While the larvae from Moorea contained more storage lipids and were more thermo-tolerant, the larvae from Moorea contained less storage lipids and were more thermo-sensitive. In this regard, it is reasonable for us to deduce that the opportunistic *S. hystrix* species stores more energy to promote their chances of survival in stressful environments, such as high-temperature environments. Additionally, the reproduction mode of brooding necessitates *S. hystrix* planula to store sufficient energy to survive long-distance dispersal [39]. Previous investigations have revealed that large planulae of *S. hystrix* have a longer lifetime, which increases their chances of finding a suitable habitat before settlement [39]. While long-chain FAs tend to participate in the synthesis of polar lipids, short-chain FAs are more readily catabolized to provide energy [40]. Therefore, the relatively highly concentrated MAG and relative short-chain FAs in *S. hystrix* may facilitate the survival of both planulae and adults in unfavorable environments. On the one hand, the relatively low-concentrated MAG and relative short-chain FAs in *P. meandrina* may be derived from the accelerated catabolism for rapid growth. Yamashiro et al. compared the lipid content and composition of *Montipora informis* in Sesoko Island, Japan between normal and tumorous tissues, and they found that the tumorous coral tissue possessed a reduced level of storage lipids for the increased energy demand for tumor synthesis [41].

In the context of global warming, it is inferred that *P. meandrina* may adapt to the environment by adjusting cell membrane structures (such as decreasing the UI of structural lipids) [19,35] or increasing storage lipid content [39]. By contrast, *S. hystrix* may reinforce competitiveness with other species by strengthening its immune system (such as synthesizing more central inflammatory modulators, including lyso-PAF) [33]. Confronted with different human activities, Scleractinia corals may also adapt to the environments by adjusting their metabolism. For Instance, Porites could adapt to increased turbidity by increasing their tissue thickness [42]. This encourages us to infer that *P. meandrina* and *S. hystrix* may acclimatize themselves to increased turbidity by storing more lipids, compensating for energy depletion due to decreased photosynthesis.

### 4.2. Resistance to Various Environmental and Biological Stresses by Small Peptides

Coral hosts or symbiotic microorganisms can synthesize peptides with a variety of biological functions, including anti-inflammatory/antioxidant [43], antibacterial [44], antiviral, defense [45], anti-fouling [46], and signal transduction properties [47]. For example, multiple antioxidant dipeptides and tripeptides were synthesized by *Montipora capitata* and *Pocillopora acuta* to defend against high-temperature stress [20]. Cyclo(L-Val-L-Pro) with antifungal activities was synthesized by the soft coral-associated fungus, *Simplicillium* sp. [44]. Small cysteine-rich peptides (SCRiPs) were found in the ectoderm of Scleractinia corals for prey capture and defense [45]. Glutathione serves as an antioxidant to participate in the oxidative stress response [48]. The higher content of multiple dendritic and cyclic dipeptides in *S. hystrix* may serve as antioxidants to resist various environmental and biological stresses in harsh conditions. Comparatively, the ideal growth environment of *P. meandrina* results in the lower production of small peptides.

### 4.3. Contributions of Other Small Molecule Metabolites on the Formation of Coral’s Life-History Traits

Aromatic amino acids, sulfhydryl compounds, and guanidyl compounds jointly participate in the regulation of the life-history traits of *P. meandrina* and *S. hystrix*. Animals are incapable of synthesizing aromatic amino acids, thus Symbiodiniaceae and bacteria in coral holobiont are responsible for their synthesis [49,50]. Aromatic amino acids possess the function of regulation of ultraviolet irradiation [51], oxidation resistance [52], and participation in secondary metabolite synthesis [53]. Previous studies showed that Symbiodiniaceae reproduction could be suppressed by the downregulation of aromatic amino acid biosynthesis [54]. Therefore, the higher content of aromatic amino acids in *P. meandrina* may contribute to its faster growth rate. On the other hand, it can counteract the harmful effect of excessive ultraviolet radiation for the shallow settlement environment. Sulfhydryl compounds possess the function of scavenging reactive oxygen species (ROS) in organisms [20]. The elevated level of 5’-methylthio-adenosine, methionine, and S-(5 ‘-adenosyl) -l-homocysteine in *P. meandrina* might reduce oxidative damage under adverse conditions, such as storm damage and excessive ultraviolet radiation. Guanidyl compounds are important nitrogen sources and relate to the microbial community composition [55,56]. The higher content of arginine, 4-guanidinobutanoic acid, and creatine in *S. hystrix* may favor its survival in nitrogen-deficient environments [57]. With the disturbance of human activities, increased nutritive salt may also change the metabolism of guanidyl compounds by reshaping microbial community structures.

## 5. Conclusions and Implications

**Figure 6:**
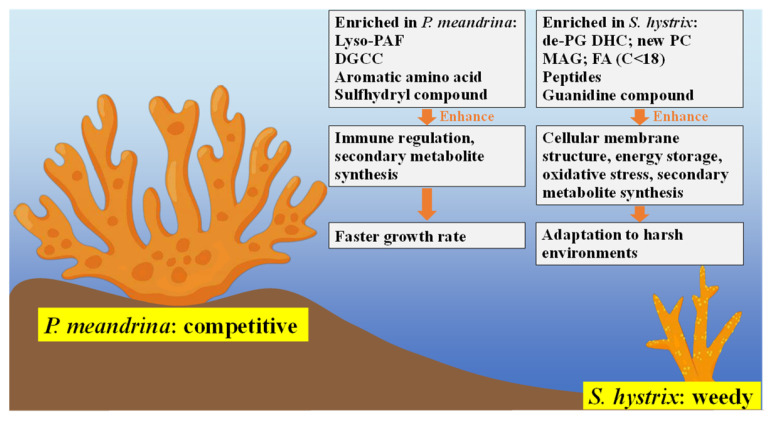
Summarized metabolic pathways involving the regulation of coral’s life-history strategy.

In this study, metabolomics were employed to characterize the physiological differences between competitive *P. meandrina* and weedy *S. hystrix*. While lyso-PAF and DGCC were more enriched in *P. meandrina*, new phospholipids, de-PG DHC, MAG and FAs (C < 18) were more enriched in *S. hystrix* (Figure 6). The content and composition profiling of lipids in *P. meandrina* and *S. hystrix* may affect their physiological traits by regulating the metabolic pathways of immune response, cell membrane structure, energy metabolism, oxidative stress system, secondary metabolite synthesis, etc. The higher content of small peptides and guanidine compounds in *S. hystrix* may aim at resisting environmental and biological stresses under adverse conditions, such as nitrogen-limited oligotrophic environments. The higher contents of aromatic amino acids and sulfhydryl compounds in *P. meandrina* may act at reducing oxidative damages from strong ultraviolet radiation and frequent storm damage in shallow seawater regions. This study contributes to understanding of the potential molecular traits underlying the life-history strategies of different coral species.

Historically, coral reef conservation has focused on passive habitat protection to alleviate the effects of various environmental stressors. However, understanding the cellular mechanisms by which corals respond to different environmental stressors may provide new insight to find ways to restore coral communities, which makes contribution to United Nations Sustainable Development Goals. Since the metabolome can reflect transient and subtle changes of cell metabolism, metabolomics has the potential to be used as a tool to sign environmental variability. For example, we can use metabolomics to explore the biochemical and physiological response mechanism of corals to acute abiotic (e.g., heat and light) and biotic (e.g., competitive interaction with other species) stresses, and to explore the differential tolerance mechanism of the same coral species from different growing habitats to environmental change, providing theoretical basis for screening resistant corals.

## Data Availability

Mass spectrometry-based metabolomics data is available in the GNPS database under the accession number MSV000089999 (https://massive.ucsd.edu/ProteoSAFe/dataset.jsp?task=a8687d9b06424602a927d77c99d3de61) (accessed on 29 July 2022).

## Supplementary Materials

The following supporting information can be downloaded at: https://www.mdpi.com/article/10.3390/metabo12111079/s1, Figure S1: Workflow diagram for exploring the potential molecular traits that contribute to the different life-history strategies of *P. meandrina* and *S. hystrix*. Figure S2: Shannon-Weiner index between the metabolite diversity of *P. meandrina* and *S. hystrix*. Figure S3: (a) OPLS-DA model for differentiating *P. meandrina* and *S. hystrix*; (b) Permutation test (*n* = 200 times) of the OPLS-DA model. Figure S4: Overview of the molecular networking of *P. meandrina* and *S. hystrix* metabolites. Figure S5: Search result of NPL_acetamidine C18:1, NPL_piperidine C18:0, NPL_pyridine C18:1 from different database (GNPS, MassBank, METLIN, ChemSpider, and Lipid-Maps). Figure S6: Mass spectra of the de-PG DHC molecular family. Figure S7: Mass spectra of the MAG molecular family. Figure S8: (a-b) Molecular networking of lyso-DGCC family and the corresponding paired sample boxplot of the signal intensities between *P. meandrina* and *S. hystrix*. (c) Mass spectra of lyso-DGCC. Figure S9: (a-b) Molecular networking of FA family and the corresponding paired sample boxplot of the signal intensities between *P. meandrina* and *S. hystrix*. (c) Mass spectra of FAs. Figure S10: Mass spectra of peptides. Figure S11: Mass spectra of small molecule metabolites.; Table S1: MZmine parameters used to extract chromatographic features. Table S2: Differential metabolites of the phospholipid family between *P. meandrina* and *S. hystrix*. Table S3: Differential metabolites of the novel phospholipid family between *P. meandrina* and *S. hystrix*. Table S4: Differential metabolites of the peptide family between *P. meandrina* and *S. hystrix*. Table S5: Differential small molecule metabolites between *P. meandrina* and *S. hystrix*.
