# Supplementary material for: Metabolomics Characterization of Scleractinia Corals with Different Life-History Strategies: A Case Study about Pocillopora meandrina and Seriatopora hystrix in the South China Sea
Source: Metabolites. 2022 Nov 8;12(11):1079. doi: 10.3390/metabo12111079 (PMC9693324; doi:10.3390/metabo12111079)
Supplement: Supplementary file 1 [file metabolites-12-01079-s001.zip › metabolites-1976043-supplementary.pdf]

Metabolomics Characterization of Scleractinia Corals with Different Life-History Strategies: A Case Study about *Pocillopora meandrina* and *Seriatopora hystrix* in the South China Sea

Jiying Pei [1], Shiguo Chen [1], Kefu Yu [1,2,*], Junjie Hu [1], Yitong Wang [1], Jingjing Zhang [1], Zhenjun Qin [1], Ruijie Zhang [1], Ting-Hao Kuo [3], Hsin-Hsiang Chung [3] and Cheng-Chih Hsu [3]

Contents

Figure S1. Workflow diagram for exploring the potential molecular traits that contribute to the different life-history strat-egies of P. meandrina and S. hystrix
Figure S2. Shannon-Weiner index between the metabolite diversity of P. meandrina and S. hystrix.
Figure S3. (a) OPLS-DA model for differentiating P. meandrina and S. hystrix; (b) Permutation test (n = 200 times) of the OPLS-DA model.
Figure S4. Overview of the molecular networking of P. meandrina and S. hystrix metabolites.
Figure S5. Search result of NPL_acetamidine C18:1, NPL_piperidine C18:0, NPL_pyridine C18:1 from different database (GNPS, MassBank, METLIN, ChemSpider, and Lipid-Maps).
Figure S6. Mass spectra of the de-PG DHC molecular family
Figure S7. Mass spectra of the MAG molecular family
Figure S8. (a-b) Molecular networking of lyso-DGCC family and the corresponding paired sample boxplot of the signal intensities between P. meandrina and S. hystrix. (c) Mass spectra of lyso-DGCC.
Figure S9. (a-b) Molecular networking of FA family and the corresponding paired sample boxplot of the signal intensities between P. meandrina and S. hystrix. (c) Mass spectra of FAs.
Figure S10. Mass spectra of peptides.
Figure S11. Mass spectra of small molecule metabolites
Table S1 MZmine parameters used to extract chromatographic features
Table S2 Differential metabolites of the phospholipid family between P. meandrina and S. hystrix.
Table S3 Differential metabolites of the novel phospholipid family between P. meandrina and S. hystrix.
Table S4 Differential metabolites of the peptide family between P. meandrina and S. hystrix.
Table S5 Differential small molecule metabolites between P. meandrina and S. hystrix.

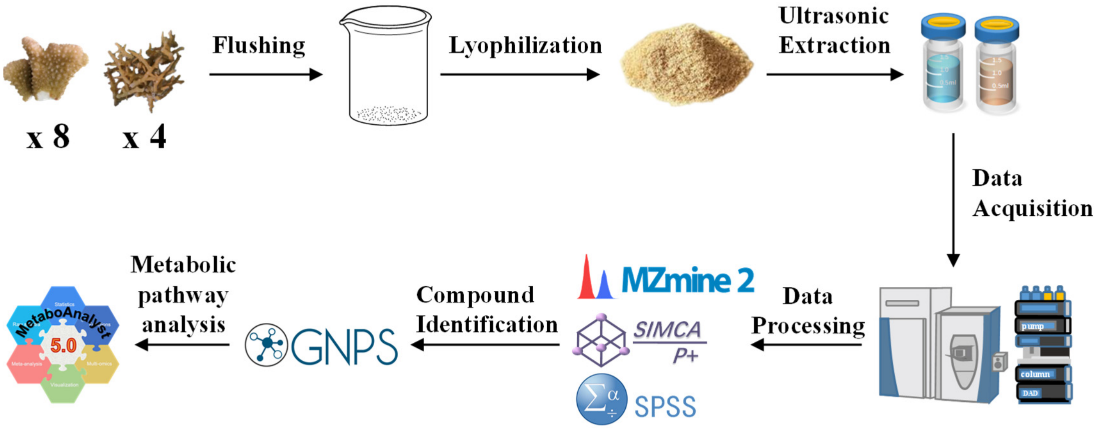

Figure S1. Workflow diagram for exploring the potential molecular traits that contribute to the different life-history strategies of *P. meandrina* and *S. hystrix*.

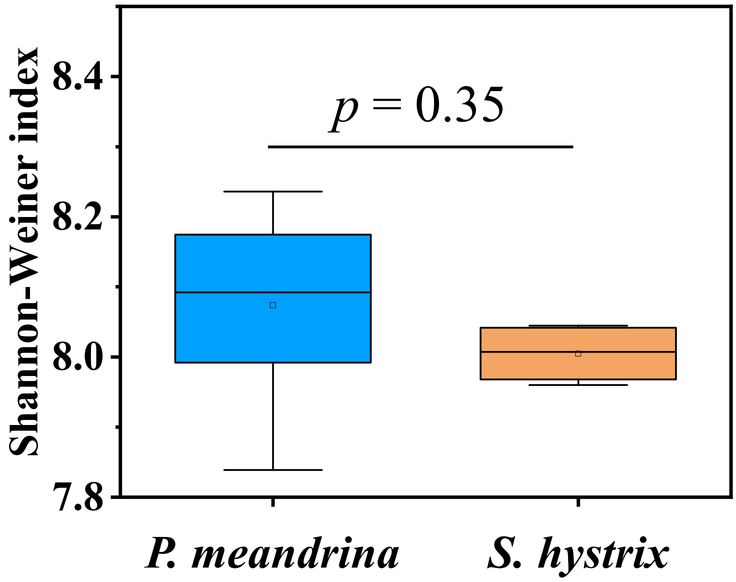

Figure S2. Shannon-Weiner index between the metabolite diversity of *P. meandrina* and *S. hystrix*.

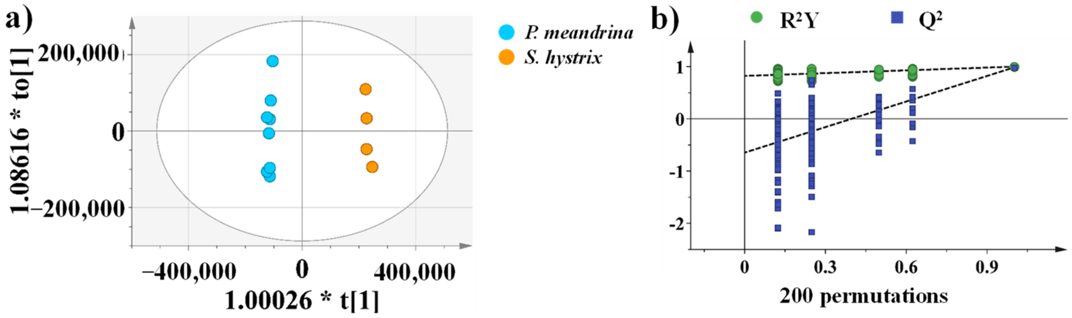

Figure S3. (a) OPLS-DA model for differentiating *P. meandrina* and *S. hystrix*; (b) Permutation test (n = 200 times) of the OPLS-DA model.

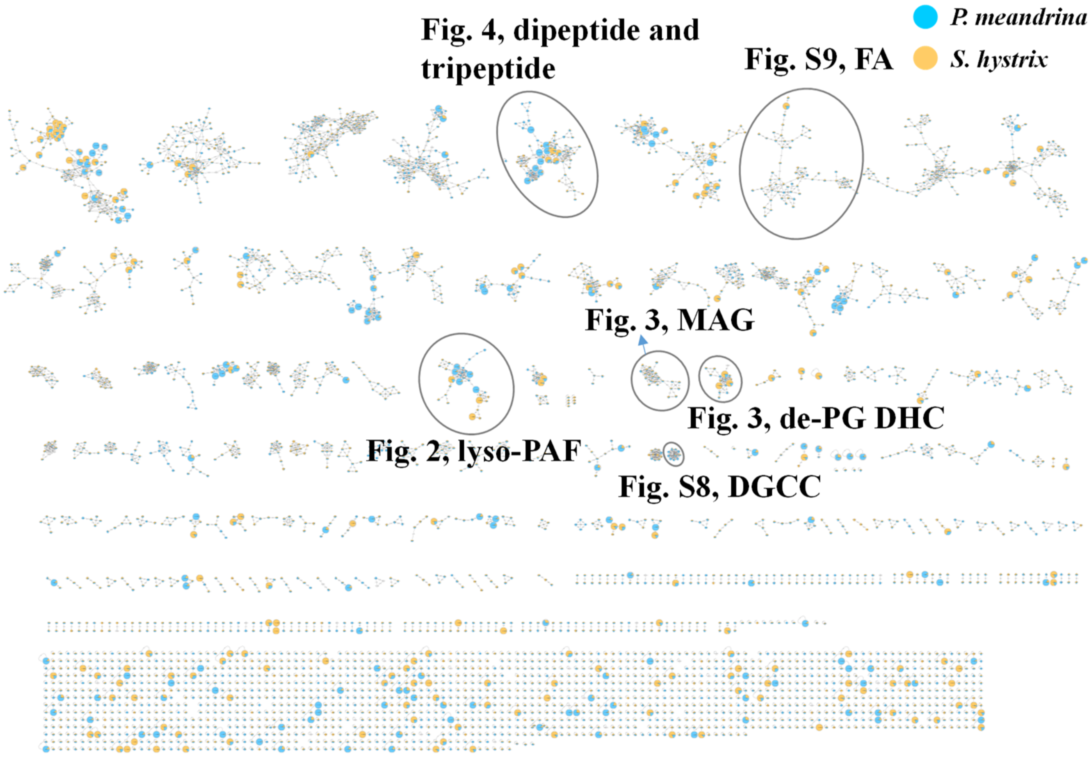

Figure S4. Overview of the molecular networking of *P. meandrina* and *S. hystrix* metabolites.

1. Search result in GNPS

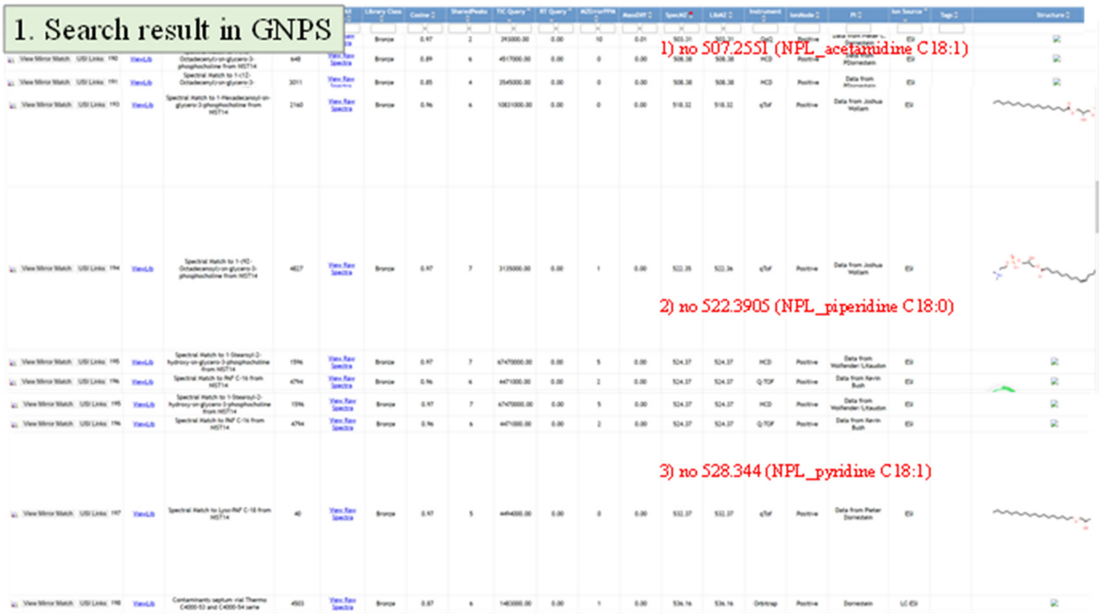

2. Search result in Massbank

MassBank Search Contents Download Accession Go Documentation About MassBank News Archive

Search Parameters :
Formula: **C25H51N2O6P** Search by $C_{25}H_{51}N_2O_6P$ (NPL_acetamidine C18:1)
Instrument Type: **EI-B , GC-EI-QQ , ESI-ITFT , ESI-QTOF , LC-ESI-ITFT , LC-ESI-QFT , LC-ESI-QQQ , APCI-ITFT , CI-B , FAB-BE , FD-B , GC-FI-TOF , LC-APCI-QTOF , MALDI-QITTOF , SI-BE EI-EBEB , GC-EI-TOF , ESI-ITTOF , ESI-TOF , LC-ESI-ITTOF , LC-ESI-QIT , LC-ESI-QTOF , APCI-ITTOF , CI-Q , FAB-EB , FI-B , LC-APCI-ITFT , LC-APPI-QQ , MALDI-TOF , GC-EI-Q CE-ESI-TOF ESI-QIT LC-ESI-IT LC-ESI-Q LC-ESI-QQ LC-ESI-TOF APCI-Q FAB-B FAB-EBEB GC-APCI-QTOF LC-APCI-Q MALDI-QIT MALDI-TOFTOF**
MS Type: **MS , MS2**
Ion Mode: **Both**

Results : 0 Hit.

Quick Search Results

MassBank Search Contents Download Accession Go Documentation About MassBank News Archive

Search Parameters :
Formula: **C27H56NO6P** Search by $C_{27}H_{56}NO_6P$ (NPL_piperidine C18:0))
Instrument Type: **EI-B , GC-EI-QQ , ESI-ITFT , ESI-QTOF , LC-ESI-ITFT , LC-ESI-QFT , LC-ESI-QQQ , APCI-ITFT , CI-B , FAB-BE , FD-B , GC-FI-TOF , LC-APCI-QTOF , MALDI-QITTOF , SI-BE EI-EBEB , GC-EI-TOF , ESI-ITTOF , ESI-TOF , LC-ESI-ITTOF , LC-ESI-QIT , LC-ESI-QTOF , APCI-ITTOF , CI-Q , FAB-EB , FI-B , LC-APCI-ITFT , LC-APPI-QQ , MALDI-TOF , GC-EI-Q CE-ESI-TOF ESI-QIT LC-ESI-IT LC-ESI-Q LC-ESI-QQ LC-ESI-TOF APCI-Q FAB-B FAB-EBEB GC-APCI-QTOF LC-APCI-Q MALDI-QIT MALDI-TOFTOF**
MS Type: **MS , MS2**
Ion Mode: **Both**

Results : 0 Hit.

Quick Search Results

MassBank Search Contents Download Accession Go Documentation About MassBank News Archive

Search Parameters :
Formula: **C28H50NO6P** Search by $C_{28}H_{50}NO_6P$ (NPL_pyridine C18:1)
Instrument Type: **EI-B , GC-EI-QQ , ESI-ITFT , ESI-QTOF , LC-ESI-ITFT , LC-ESI-QFT , LC-ESI-QQQ , APCI-ITFT , CI-B , FAB-BE , FD-B , GC-FI-TOF , LC-APCI-QTOF , MALDI-QITTOF , SI-BE EI-EBEB , GC-EI-TOF , ESI-ITTOF , ESI-TOF , LC-ESI-ITTOF , LC-ESI-QIT , LC-ESI-QTOF , APCI-ITTOF , CI-Q , FAB-EB , FI-B , LC-APCI-ITFT , LC-APPI-QQ , MALDI-TOF , GC-EI-Q CE-ESI-TOF ESI-QIT LC-ESI-IT LC-ESI-Q LC-ESI-QQ LC-ESI-TOF APCI-Q FAB-B FAB-EBEB GC-APCI-QTOF LC-APCI-Q MALDI-QIT MALDI-TOFTOF**
MS Type: **MS , MS2**
Ion Mode: **Both**

Results : 0 Hit.

3. Search result in METLIN

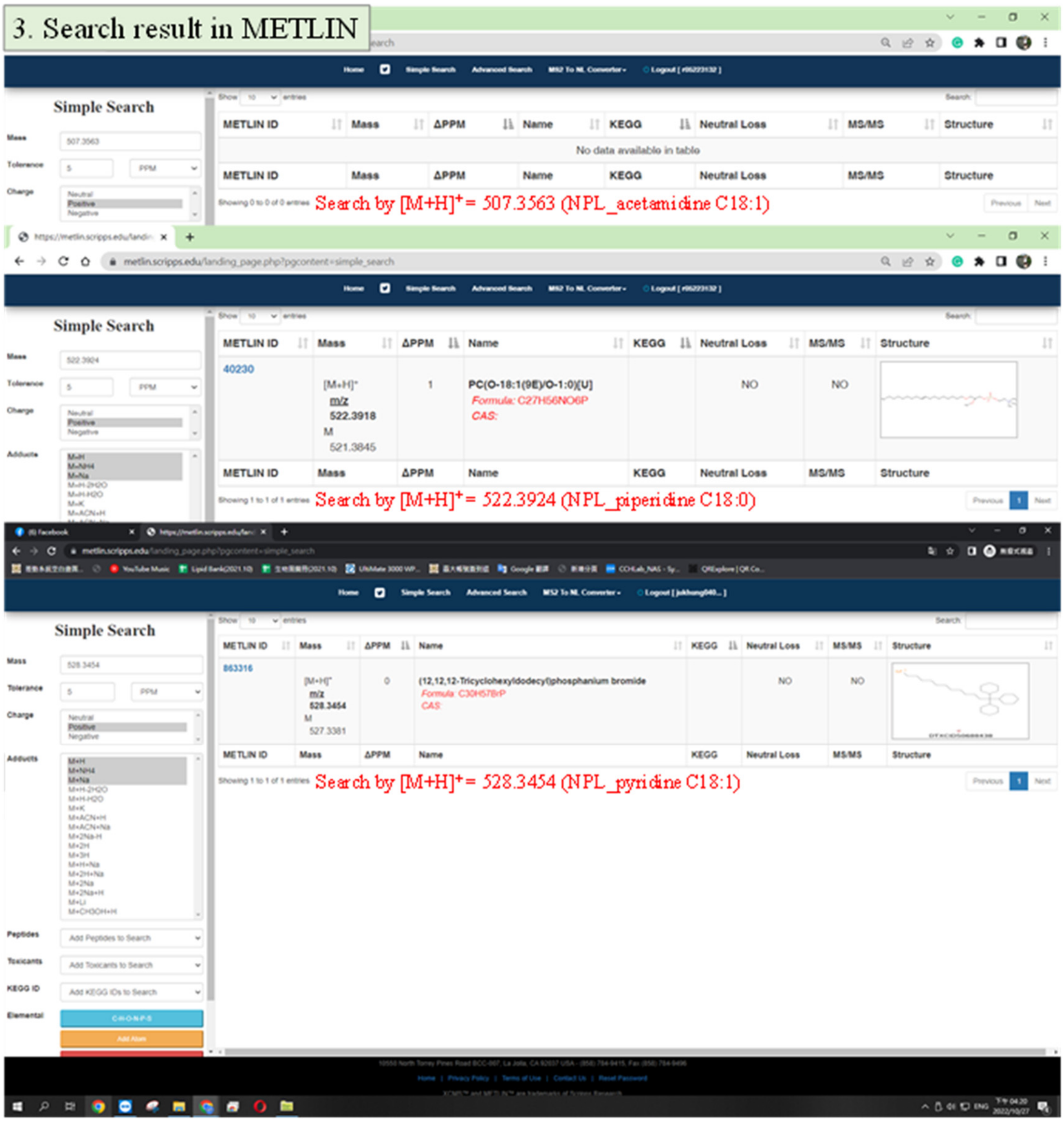

4. Search result in Chemspider

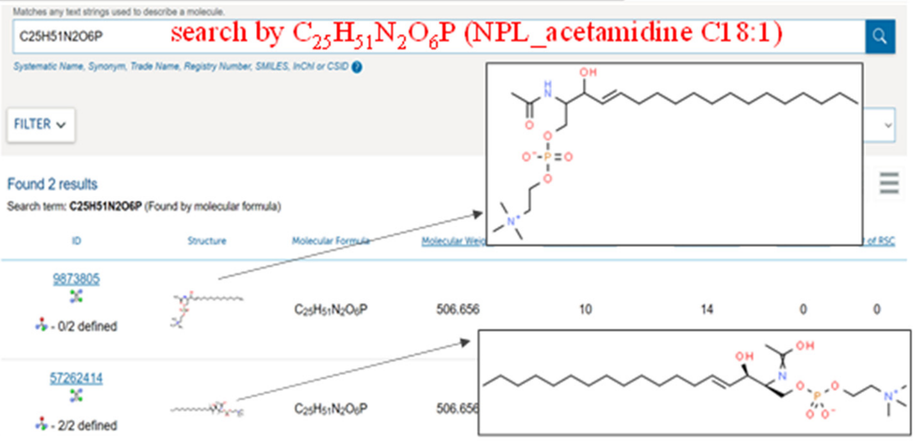

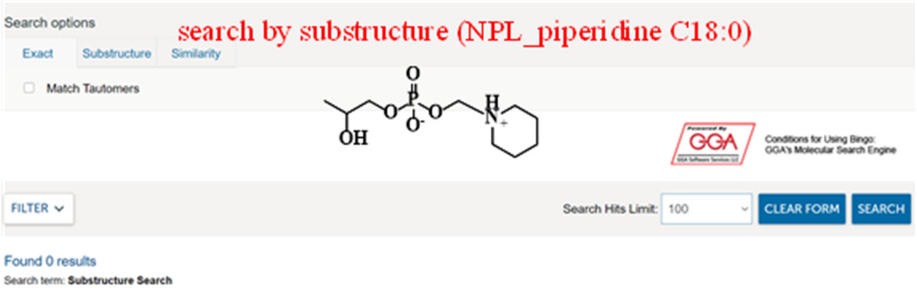

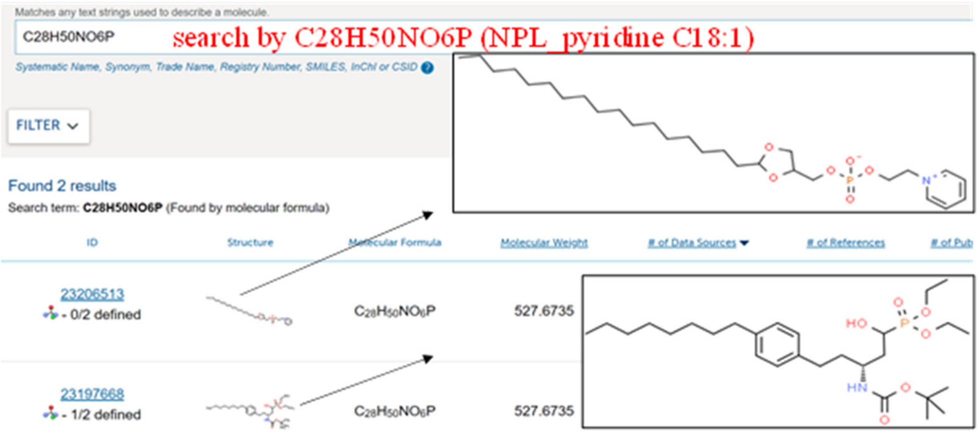

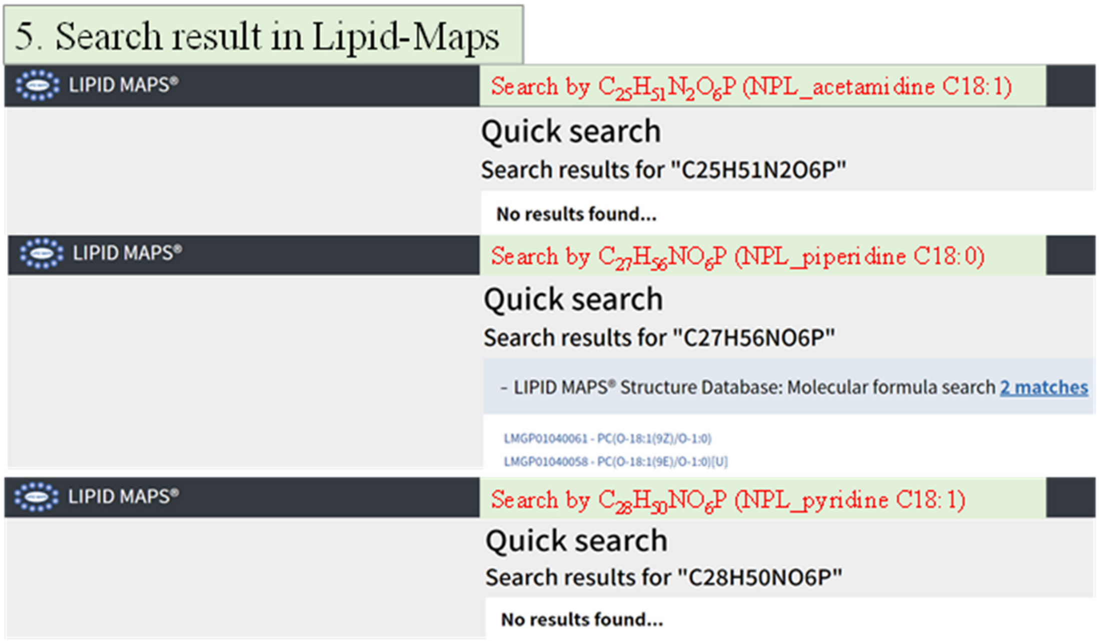

Figure S5. Search result of NPL_acetamidine C18:1, NPL_piperidine C18:0, NPL_pyridine C18:1 from different database (GNPS, MassBank, METLIN, ChemSpider, and Lipid-Maps).

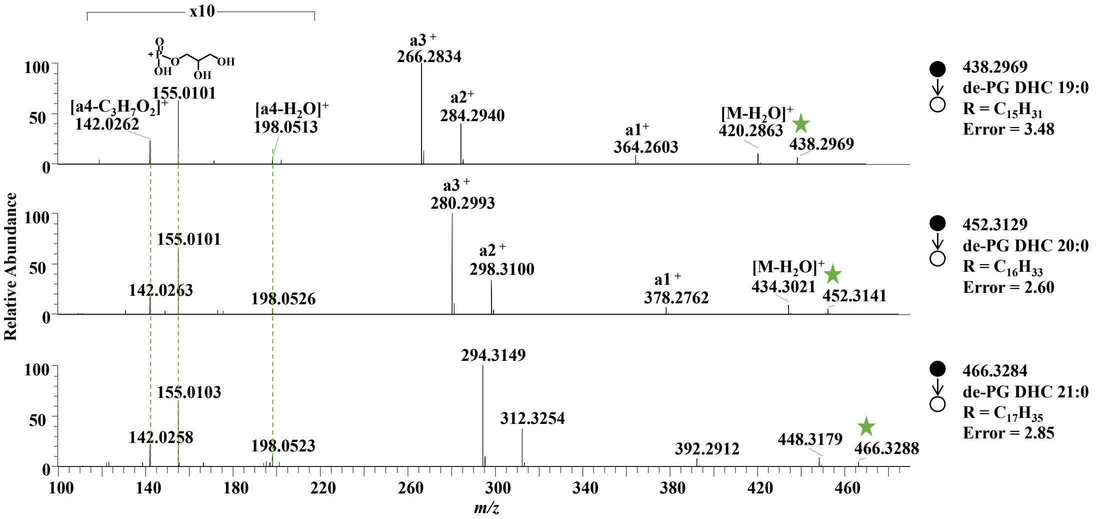

Figure S6. Mass spectra of the de-PG DHC molecular family. (The green asterisk means the parent ion of the MS/MS spectrum.)

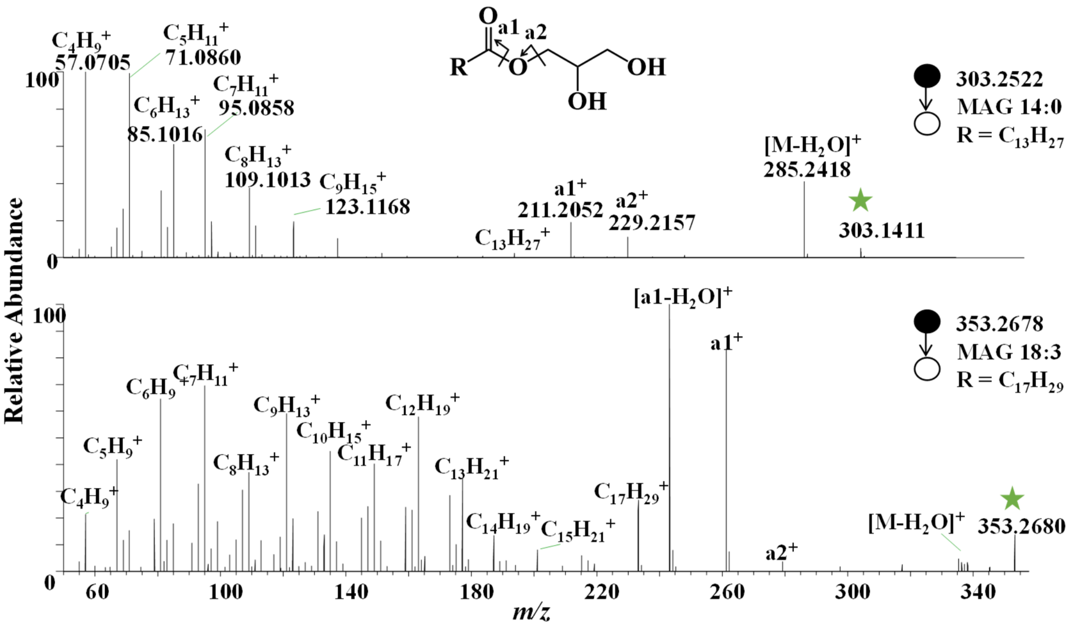

Figure S7. Mass spectra of the MAG molecular family. (The green asterisk means the parent ion of the MS/MS spectrum.)

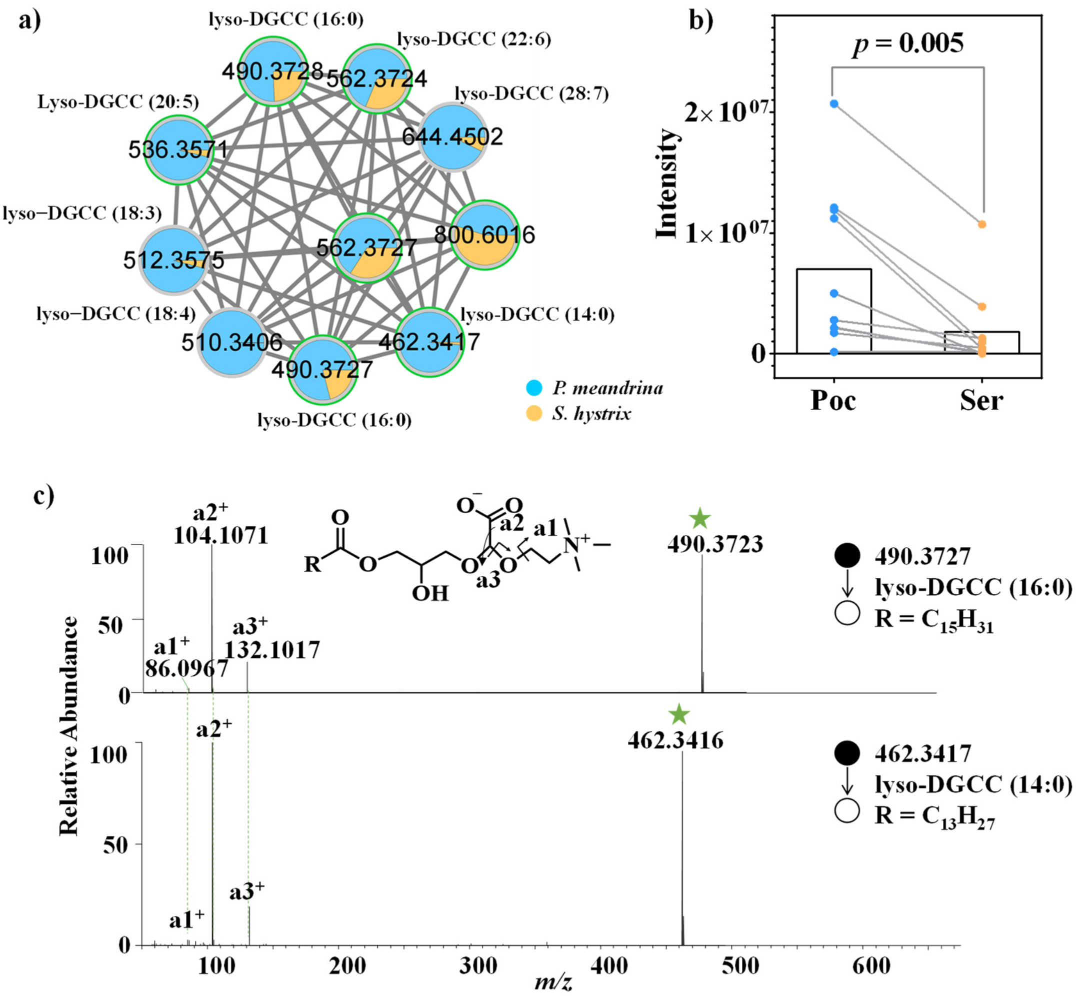

Figure S8. (a-b) Molecular networking of lyso-DGCC family and the corresponding paired sample boxplot of the signal intensities between *P. meandrina* and *S. hystrix*. (c) Mass spectra of lyso-DGCC. (The green asterisk means the parent ion of the MS/MS spectrum.)

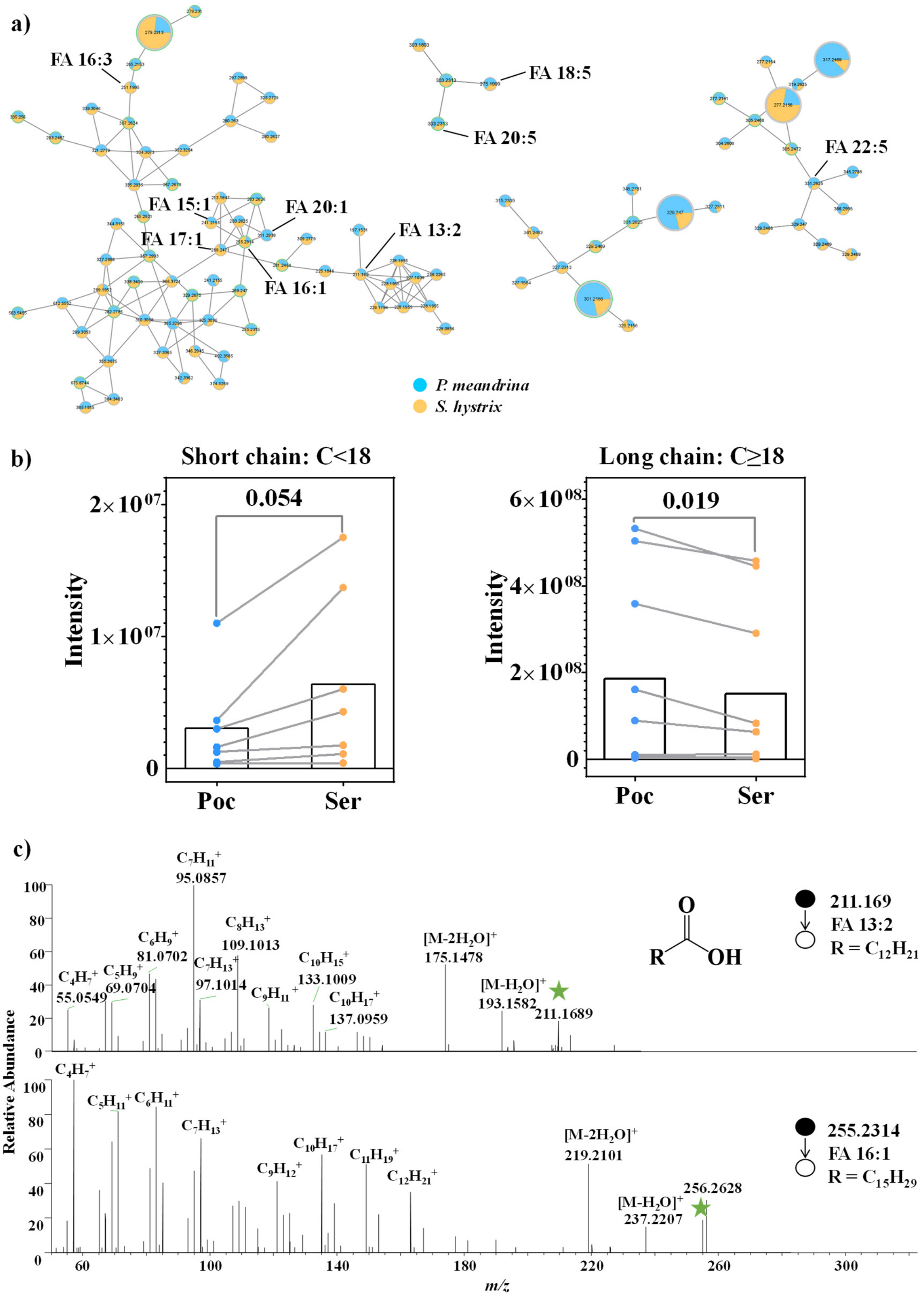

Figure S9. (a-b) Molecular networking of FA family and the corresponding paired sample boxplot of the signal intensities between *P. meandrina* and *S. hystrix*. (c) Mass spectra of FAs. (The green asterisk means the parent ion of the MS/MS spectrum.)

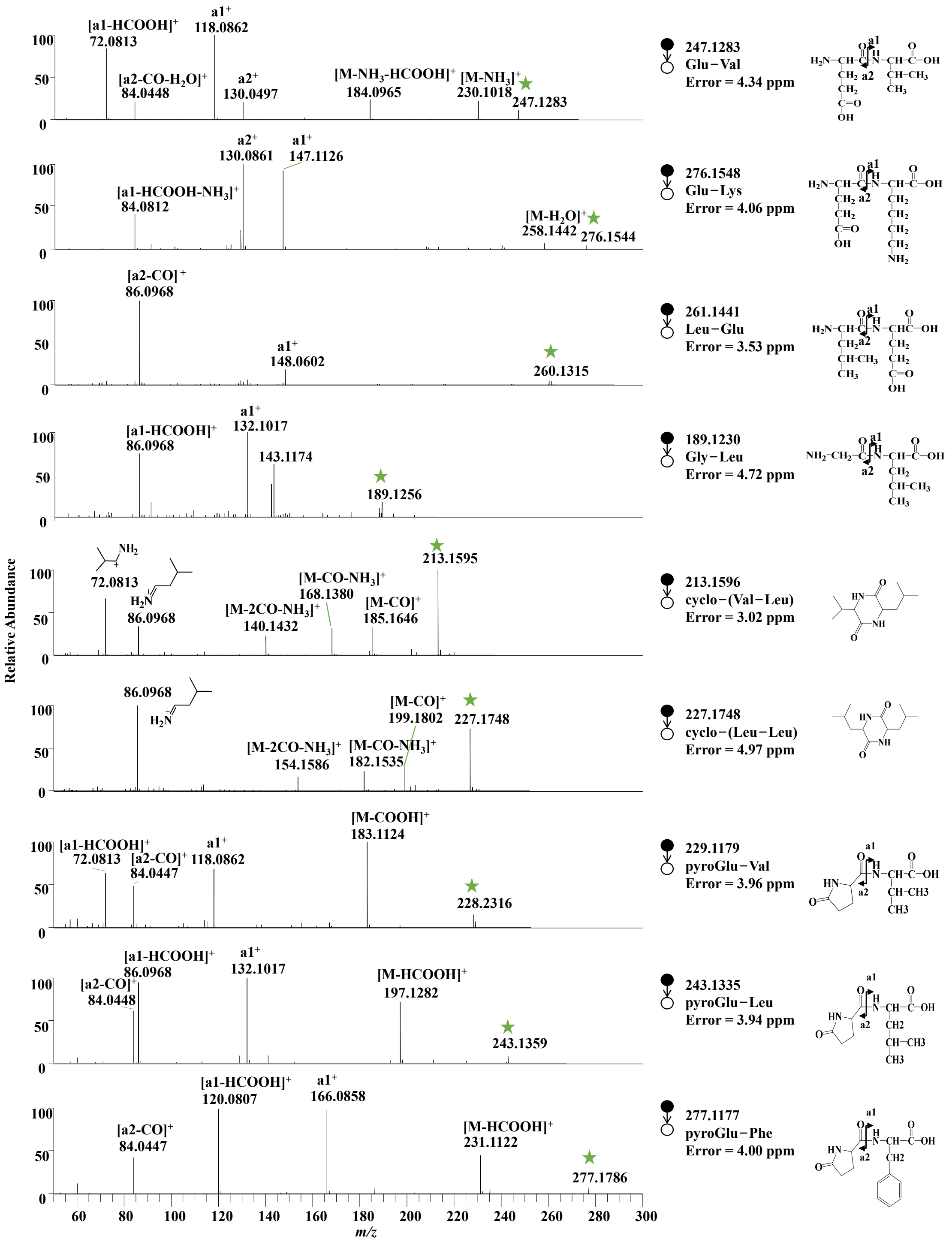

Figure S10. Mass spectra of peptides. (The green asterisk means the parent ion of the MS/MS spectrum.)

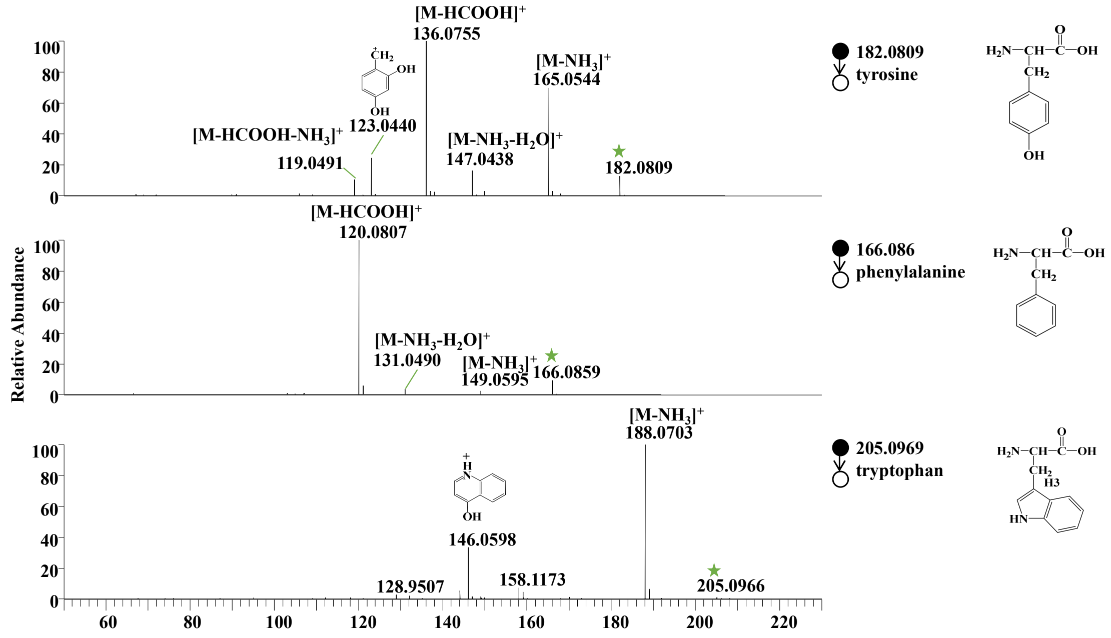
[M-HCOOH]+
136.0755
[M-NH3]+
165.0544
[M-HCOOH-NH3]+
123.0440
119.0491
[M-NH3-H2O]+
147.0438
182.0809
182.0809
tyrosine
[M-HCOOH]+
120.0807
Relative Abundance
[M-NH3-H2O]+
131.0490
[M-NH3]+
149.0595
166.0859
166.086
phenylalanine
[M-NH3]+
188.0703
146.0598
128.9507
158.1173
205.0966
205.0969
tryptophan
60
80
100
120
140
160
180
200
220

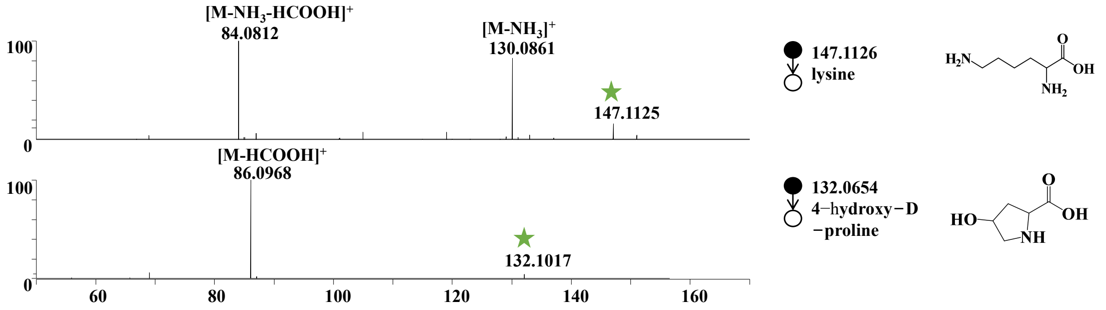
[M-NH3-HCOOH]+
84.0812
[M-NH3]+
130.0861
147.1125
147.1126
lysine
[M-HCOOH]+
86.0968
132.1017
132.0654
4−hydroxy−D
−proline
60
80
100
120
140
160

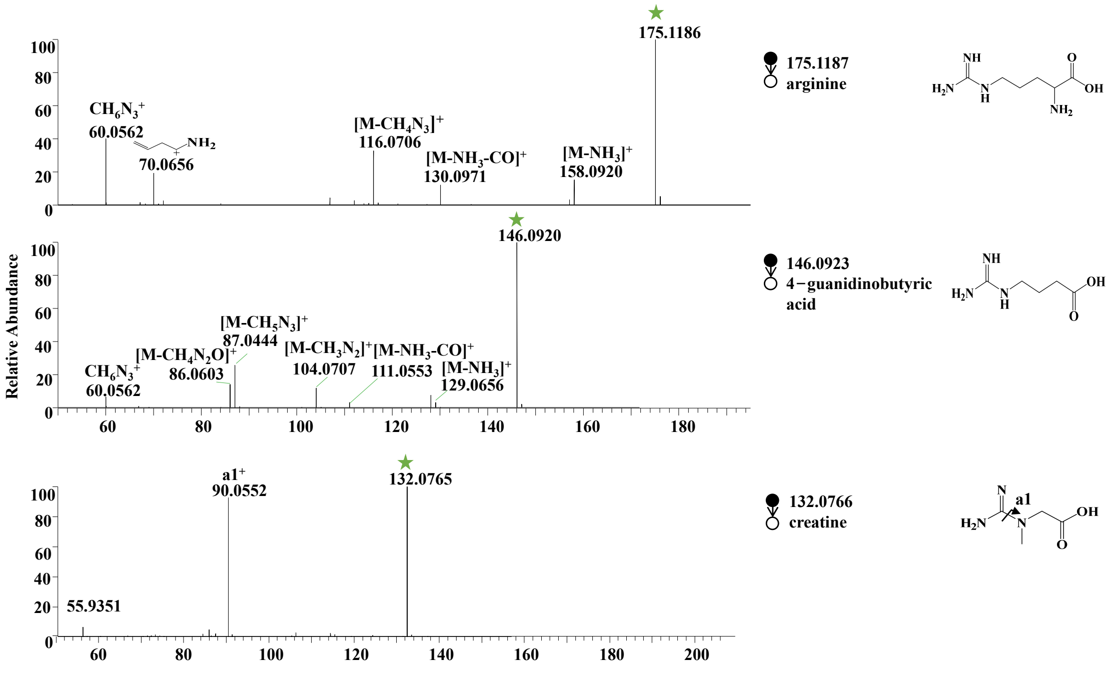
175.1186
175.1187
arginine
CH6N3+
60.0562
70.0656
[M-CH4N3]+
116.0706
[M-NH3-CO]+
130.0971
[M-NH3]+
158.0920
146.0920
146.0923
4−guanidinobutyric
acid
Relative Abundance
[M-CH5N3]+
87.0444
[M-CH4N2O]+
86.0603
CH6N3+
60.0562
[M-CH3N2]+
104.0707
[M-NH3-CO]+
111.0553
[M-NH3]+
129.0656
a1+
90.0552
132.0765
55.9351
132.0766
creatine
60
80
100
120
140
160
180
200

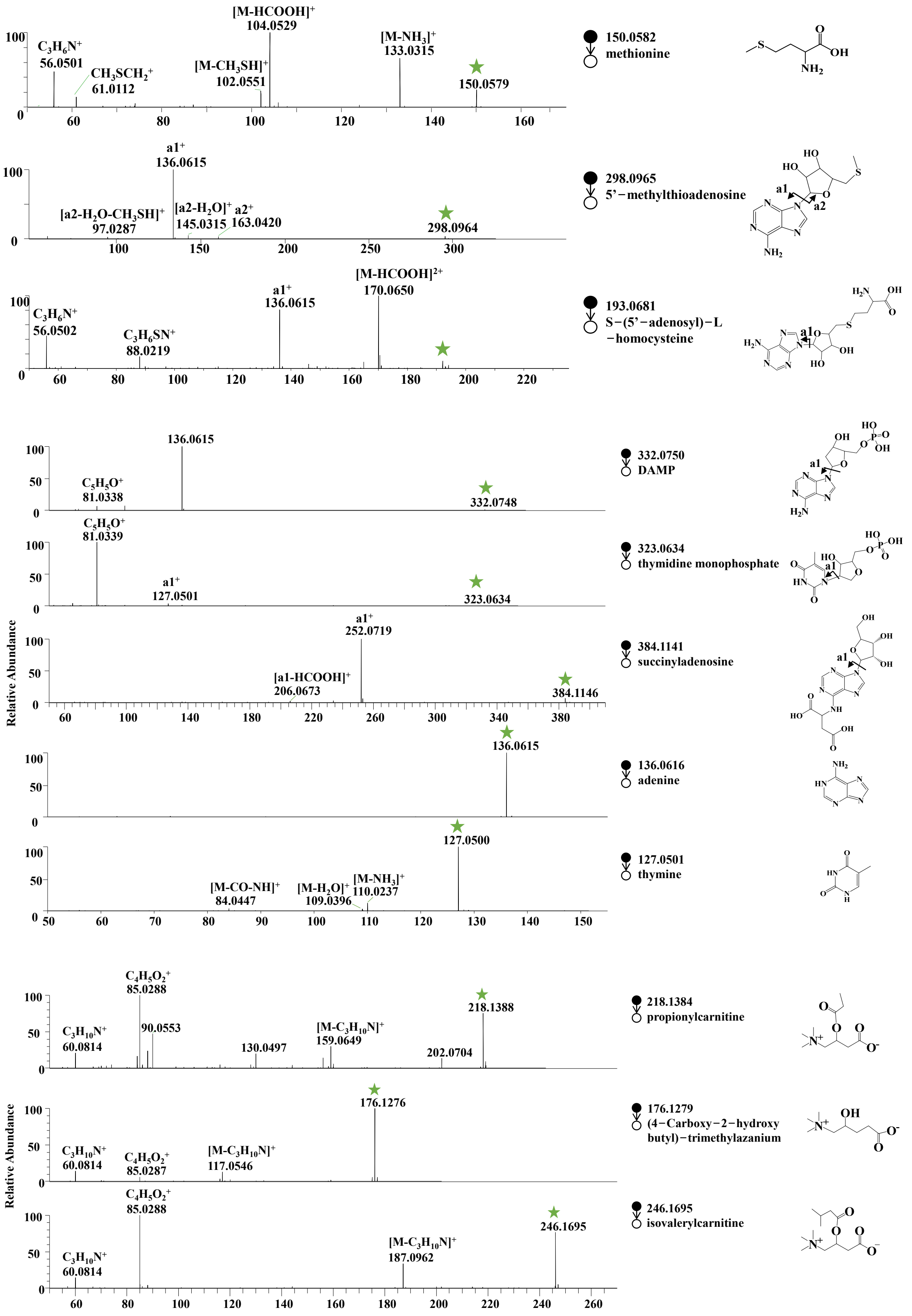
[M-HCOOH]+ 104.0529
[M-NH3]+ 133.0315
C3H6N+ 56.0501
CH3SCH2+ 61.0112
[M-CH3SH]+ 102.0551
150.0579
150.0582
methionine
a1+ 136.0615
[a2-H2O-CH3SH]+ 97.0287
[a2-H2O]+ 145.0315
a2+ 163.0420
298.0964
298.0965
5’-methylthioadenosine
[M-HCOOH]2+ 170.0650
a1+ 136.0615
C3H6N+ 56.0502
C3H6SN+ 88.0219
193.0681
S-(5’-adenosyl)-L-homocysteine
136.0615
C5H5O+ 81.0338
332.0748
332.0750
DAMP
C5H5O+ 81.0339
a1+ 127.0501
323.0634
323.0634
thymidine monophosphate
a1+ 252.0719
[a1-HCOOH]+ 206.0673
384.1146
384.1141
succinyladenosine
136.0615
136.0616
adenine
127.0500
[M-CO-NH]+ 84.0447
[M-H2O]+ 109.0396
[M-NH3]+ 110.0237
127.0501
thymine
Relative Abundance
C4H5O2+ 85.0288
C3H10N+ 60.0814
90.0553
130.0497
[M-C3H10N]+ 159.0649
202.0704
218.1388
218.1384
propionylcarnitine
176.1276
C3H10N+ 60.0814
C4H5O2+ 85.0287
[M-C3H10N]+ 117.0546
176.1279
(4-Carboxy-2-hydroxy butyl)-trimethylazanium
C4H5O2+ 85.0288
C3H10N+ 60.0814
[M-C3H10N]+ 187.0962
246.1695
246.1695
isovalerylcarnitine

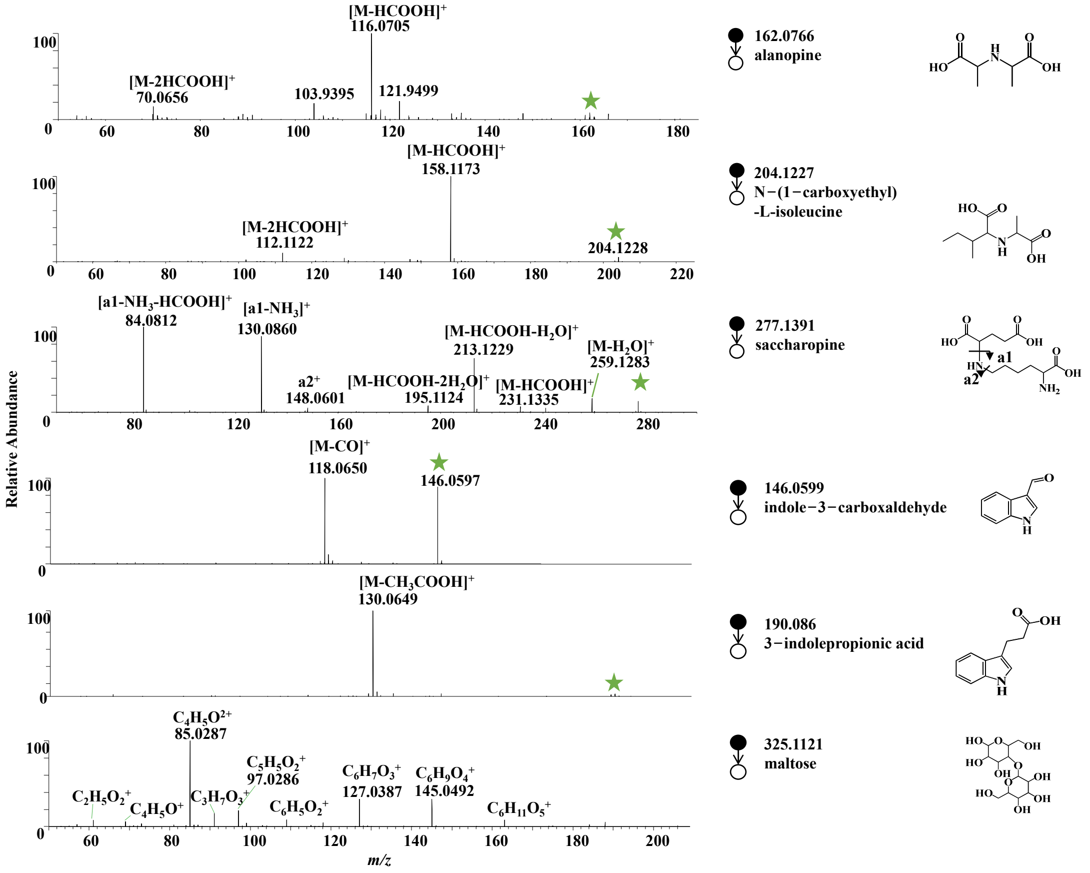

Figure S11. Mass spectra of small molecule metabolites. (The green asterisk means the parent ion of the MS/MS spectrum.)

Table S1. MZmine parameters used to extract chromatographic features.

Steps	Module	Parameters
1. MS1 Mass detection	Exact mass	• Retention time (RT): 0.5-28 min<br>• Noise level: 7.0E4
2. MS2 Mass detection	Exact mass	• RT: 0.5-28 min<br>• Noise level: 1.0E4
3. Chromatogram builder	ADAP chromatogram builder	• Min group size of scans: 5<br>• Group intensity threshold: 7.0E3<br>• Min highest intensity: 7.0E4<br>• *m/z* tolerance: 0.001 Da or 5 ppm
4. Chromatogram deconvolution	Local minimum search	• Chromatographic threshold: 95%<br>• Search min in RT range: 0.1 min<br>• Min relative height: 3.0%<br>• Min absolute height: 7.0E4<br>• Min ratio of peak top/edge: 2<br>• Peak duration range: 0.07-1.20 min
5. Isotopic peak grouper		• *m/z* tolerance: 0.003 Da or 12 ppm<br>• RT tolerance: 0.05 min
6. Join aligner		• *m/z* tolerance: 0.001 Da or 5 ppm<br>• RT tolerance: 0.15 min<br>• Weight for *m/z*: 75%<br>• Weight for RT: 25%
7. Feature list rows filter		• Min peaks in a row: 6
8. Peak finder (multithreaded)		• Intensity tolerance: 5%<br>• *m/z* tolerance: 0.001 Da or 5 ppm<br>• RT tolerance: 0.15 min
9. Duplicate peak filter		• *m/z* tolerance: 0.002 Da or 5 ppm<br>• RT tolerance: 0.2 min
10. Adduct search		• *m/z* tolerance: 0.002 Da or 5 ppm<br>• RT tolerance: 0.2 min
11. Fragment search		• *m/z* tolerance: 0.001 Da or 5 ppm<br>• RT tolerance: 0.05 min<br>• Max fragment peak height: 50%<br>• Min MS2 peak height: 3.0E4

Table S2. Differential metabolites of the phospholipid family between *P. meandrina* and *S. hystrix*.

ID	VIP	Name	Adduct	*m/z*	RT/min	Error/ppm	Molecular Formula	*p*	FC	Pie Chart
15	8.82	lyso-PAF C-16	$[M+H]^+$	482.3592	24.60	3.79	$C_{24}H_{52}NO_6P$	0.009	19.37	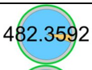
26	7.27	lyso-PAF C-18	$[M+H]^+$	510.3907	25.84	3.19	$C_{26}H_{56}NO_6P$	0.019	9.90	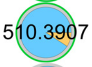
37	7.22	lyso-PAF C-16	$[M+Na]^+$	504.341	24.60	4.02	$C_{24}H_{51}NO_6PNa$	0.002	17.26	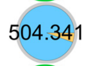
40	6.47	lyso-PAF C-18	$[M+Na]^+$	532.3725	25.84	3.43	$C_{26}H_{55}NO_6PNa$	0.007	7.94	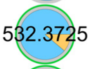
338	2.45	lyso PC P-16:0	$[M+H]^+$	480.3438	23.84	3.28	$C_{24}H_{50}NO_6P$	0.000	10.34	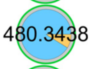
486	2.05	lyso PC P-16:0	$[M+Na]^+$	502.3253	23.84	4.13	$C_{24}H_{49}NO_6PNa$	0.001	12.42	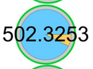
648	1.34	lyso PC P-18:0	$[M+H]^+$	508.3752	25.60	2.91	$C_{26}H_{54}NO_6P$	0.049	31.83	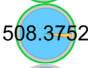
1042	1.03	lyso-PAF C-20	$[M+H]^+$	538.4219	27.44	3.21	$C_{28}H_{60}NO_6P$	0.021	14.43	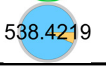

Table S3. Differential metabolites of the novel phospholipid family between *P. meandrina* and *S. hystrix*.

ID	VIP>1	Name	Adduct	*m/z*	RT/min	Error/ppm	Molecular Formula	*p*	FC	Pie Chart
5451	1.87	NPL_pyridine C18:1	$[M+H]^+$	528.344	25.80	2.61	$C_{28}H_{50}NO_6P$	0.004	0.01	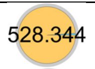
5468	1.51	NPL_pyridine C18:1	$[M+Na]^+$	550.3261	25.81	2.32	$C_{28}H_{50}NO_6P$	0.003	0.01	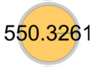
4884	<1	NPL_pyridine C15:1	$[M+H]^+$	500.3123	24.47	3.55	$C_{26}H_{46}NO_6P$	0.019	0.14	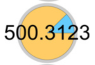
5344	<1	NPL_pyridine C15:1	$[M+Na]^+$	522.294	24.47	3.98	$C_{26}H_{45}NO_6PNa$	0.017	0.11	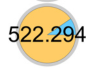
1154	<1	NPL_pyridine C18:1	$[M+H]^+$	528.3436	25.57	3.36	$C_{28}H_{50}NO_6P$	0.138	0.36	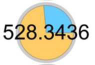
1806	<1	NPL_pyridine C18:1	$[M+Na]^+$	550.3254	25.57	3.59	$C_{28}H_{49}NO_6PNa$	0.093	0.30	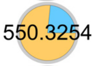
3971	<1	NPL_piperidine C16:0	$[M+H]^+$	494.3588	24.61	4.45	$C_{25}H_{52}NO_6P$	0.826	1.13	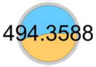
2151	<1	NPL_piperidine C16:0	$[M+Na]^+$	516.3413	24.61	3.29	$C_{25}H_{51}NO_6PNa$	0.431	0.67	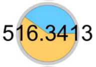
6201	<1	NPL_piperidine C18:0	$[M+H]^+$	522.3905	25.87	3.64	$C_{27}H_{56}NO_6P$	0.156	0.48	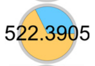
2629	<1	NPL_piperidine C18:0	$[M+Na]^+$	544.373	25.86	2.57	$C_{27}H_{55}NO_6PNa$	0.068	0.37	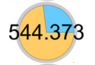
4841	<1	NPL_acetamidine C18:1	$[M+H]^+$	507.3551	25.62	3.35	$C_{25}H_{51}N_2O_6P$	0.093	0.42	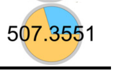

Table S4. Differential metabolites of the peptide family between *P. meandrina* and *S. hystrix*.

ID	VIP	Name	Adduct	*m/z*	RT/min	Error/ppm	Molecular Formula	*p*	FC	Pie Chart
1263	<1	Glu-Val	$[M+H]^+$	247.1283	5.94	4.34	$C_{10}H_{18}N_2O_5$	0.125	0.56	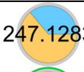
2816	<1	Glu-Lys	$[M+H]^+$	276.1548	1.28	4.06	$C_{11}H_{21}N_3O_5$	0.007	0.41	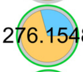
5454	<1	Leu-Glu	$[M+H]^+$	261.1441	1.72	3.53	$C_{11}H_{20}N_2O_5$	0.000	0.14	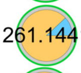
5410	<1	Gly-Leu	$[M+H]^+$	189.1228	3.80	5.78	$C_8H_{16}N_2O_3$	0.000	0.06	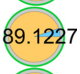
5491	<1	cyclo-(Val-Leu)	$[M-H_2O+H]^+$	213.1596	15.06	3.42	$C_{11}H_{22}N_2O_3$	0.000	0.02	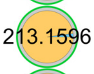
5521	<1	cyclo-(Leu-Leu)	$[M+H]^+$	227.1748	16.41	4.97	$C_{12}H_{22}N_2O_2$	0.000	0.03	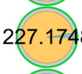
3073	<1	PyroGlu-Val	$[M+H]^+$	229.1179	9.92	3.96	$C_{10}H_{16}N_2O_4$	0.050	0.60	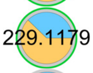
1068	<1	PyroGlu-Leu	$[M+H]^+$	243.1335	12.47	3.94	$C_{11}H_{18}N_2O_4$	0.044	0.61	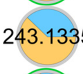
3284	<1	PyroGlu-Phe	$[M+H]^+$	277.1177	13.26	4.00	$C_{14}H_{16}N_2O_4$	0.001	0.40	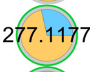
1446	<1	Glu-Phe	$[M+H]^+$	295.1283	10.93	3.64	$C_{14}H_{18}N_2O_5$	0.036	2.17	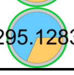

Table S5. Differential small molecule metabolites between *P. meandrina* and *S. hystrix*.

ID	VIP	Name	*m/z*	RT/min	Error/ppm	Molecular Formula	*p*	FC	Pie Chart
				Amino acids					
170	2.75	Tyrosine	182.0809	3.50	4.36	$C_9H_{11}NO_3$	0.042	3.5	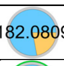
94	9.19	Phenylalanine	166.0860	5.06	4.69	$C_9H_{11}NO_2$	0.011	4.8	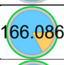
545	2.98	Tryptophan	205.0969	8.31	3.79	$C_{11}H_{12}N_2O_2$	0.011	7.5	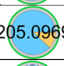
75	5.54	Lysine	147.1126	1.15	4.95	$C_6H_{14}N_2O_2$	0.033	2.30	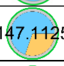
212	2.05	4-Hydroxy-D-proline	132.0654	1.53	4.88	$C_5H_9NO_3$	0.000	4.10	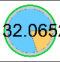
				Guanidyl compounds					
483	5.22	Arginine	175.1186	1.22	5.00	$C_6H_{14}N_4O_2$	0.005	0.30	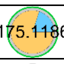
341	5.88	4-Guanidinobutyric acid	146.0922	1.36	4.98	$C_5H_{11}N_3O_2$	0.000	0.10	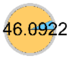
540	7.13	Creatine	132.0766	1.45	5.27	$C_4H_9N_3O_2$	0.000	0.09	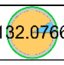
				Sulfhydryl compounds					
304	1.63	S-(5'-Adenosyl)-L-homocysteine	193.0681 $[M+2H]^{2+}$	1.71	2.68	$C_{14}H_{20}N_6O_5S$	0.000	5.18	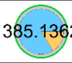
55	4.11	Methionine	150.0582	1.73	4.34	$C_5H_{11}NO_2S$	0.024	2.40	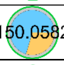
348	2.18	5'-Methylthioadenosine	298.0965	9.31	2.89	$C_{11}H_{15}N_5O_3S$	0.006	5.31	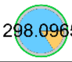
				Base/nucleotide					
1161	1.17	DAMP	332.0750	1.78	2.93	$C_{10}H_{14}N_5O_6P$	0.001	5.61	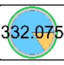
2172	1.12	Thymidine monophosphate	323.0634	8.64	3.11	$C_{10}H_{15}N_2O_8P$	0.000	7.81	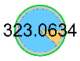
1205	1.11	Succinyl-adenosine	384.1141	9.80	3.68	$C_{14}H_{17}N_5O_8$	0.000	4.09	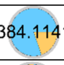
268	4.76	Adenine	136.0616	1.46	5.11	$C_5H_5N_5$	0.023	0.32	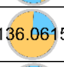
332	5.19	Thymine	127.0501	3.78	4.94	$C_5H_6N_2O_2$	0.001	0.46	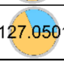
				Other small molecules					
240	1.63	Propionyl-carnitine	218.1384	1.71	3.71	$C_{10}H_{19}NO_4$	0.002	4.06	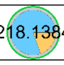
209	6.02	(4-Carboxy-2-hydroxybutyl)-trimethylazanium	176.1279	1.39	4.37	$C_8H_{18}NO_3$	0.001	0.21	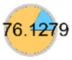
1634	1.18	Isovaleryl-carnitine	246.1695	7.97	4.10	$C_{12}H_{23}NO_4$	0.003	0.39	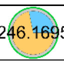
512	2.68	N-(1-Carboxyethyl)-L-isoleucine	204.1227	8.95	4.05	$C_9H_{17}NO_4$	0.006	134.3	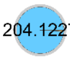
858	1.62	Alanopine	162.0758	2.48	4.99	$C_6H_{11}NO_4$	0.000	65.4	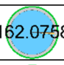
1810	2.75	Indole-3-Carboxaldehyde	146.0599	13.82	4.60	C_9H_7NO	0.000	0.05	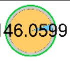
5446	1.29	3-Indolepropionic acid	190.086	16.40	4.21	$C_{11}H_{11}NO_2$	0.000	0.00	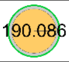
775	1.95	Saccharopine	277.1391	1.50	3.25	$C_{11}H_{20}N_2O_6$	0.000	0.19	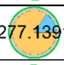
1295	1.11	Maltose	325.1121 $[M-H_2O+H]^+$	1.63	4.12	$C_{12}H_{22}O_{11}$	0.000	0.39	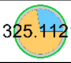
